# Ferroptosis-Regulated Natural Products and miRNAs and Their Potential Targeting to Ferroptosis and Exosome Biogenesis

**DOI:** 10.3390/ijms25116083

**Published:** 2024-05-31

**Authors:** Ya-Ting Chuang, Ching-Yu Yen, Tsu-Ming Chien, Fang-Rong Chang, Yi-Hong Tsai, Kuo-Chuan Wu, Jen-Yang Tang, Hsueh-Wei Chang

**Affiliations:** 1 Department of Biomedical Science and Environmental Biology, PhD Program in Life Sciences, College of Life Science, Kaohsiung Medical University, Kaohsiung 80708, Taiwan; u112851002@gap.kmu.edu.tw; 2 School of Dentistry, Taipei Medical University, Taipei 11031, Taiwan; ycy@tmu.edu.tw; 3 Department of Oral and Maxillofacial Surgery, Chi-Mei Medical Center, Tainan 71004, Taiwan; 4 Department of Urology, Kaohsiung Medical University Hospital, Kaohsiung 80708, Taiwan; u108801005@kmu.edu.tw; 5 School of Post-Baccalaureate Medicine, Kaohsiung Medical University, Kaohsiung 80708, Taiwan; 6 Department of Urology, Kaohsiung Gangshan Hospital, Kaohsiung Medical University, Kaohsiung 820111, Taiwan; 7 Graduate Institute of Natural Products, Kaohsiung Medical University, Kaohsiung 80708, Taiwan; aaronfrc@kmu.edu.tw; 8 Department of Pharmacy and Master Program, College of Pharmacy and Health Care, Tajen University, Pingtung 907101, Taiwan; bear@tajen.edu.tw; 9 Department of Computer Science and Information Engineering, National Pingtung University, Pingtung 900391, Taiwan; kcwu@nptu.edu.tw; 10 Department of Radiation Oncology, Kaohsiung Medical University Hospital, Kaohsiung Medical University, Kaohsiung 80708, Taiwan; 11 Department of Medical Research, Kaohsiung Medical University Hospital, Kaohsiung 80708, Taiwan; 12 Center for Cancer Research, Kaohsiung Medical University, Kaohsiung 80708, Taiwan

**Keywords:** exosome, miRNA, ferroptosis, exosome biogenesis, natural products

## Abstract

Ferroptosis, which comprises iron-dependent cell death, is crucial in cancer and non-cancer treatments. Exosomes, the extracellular vesicles, may deliver biomolecules to regulate disease progression. The interplay between ferroptosis and exosomes may modulate cancer development but is rarely investigated in natural product treatments and their modulating miRNAs. This review focuses on the ferroptosis-modulating effects of natural products and miRNAs concerning their participation in ferroptosis and exosome biogenesis (secretion and assembly)-related targets in cancer and non-cancer cells. Natural products and miRNAs with ferroptosis-modulating effects were retrieved and organized. Next, a literature search established the connection of a panel of ferroptosis-modulating genes to these ferroptosis-associated natural products. Moreover, ferroptosis-associated miRNAs were inputted into the miRNA database (miRDB) to bioinformatically search the potential targets for the modulation of ferroptosis and exosome biogenesis. Finally, the literature search provided a connection between ferroptosis-modulating miRNAs and natural products. Consequently, the connections from ferroptosis–miRNA–exosome biogenesis to natural product-based anticancer treatments are well-organized. This review sheds light on the research directions for integrating miRNAs and exosome biogenesis into the ferroptosis-modulating therapeutic effects of natural products on cancer and non-cancer diseases.

## 1. Introduction

### 1.1. Relationship between Exosomes and Ferroptosis

Exosomes are extracellular vesicles with a nanometer-scale size (30–150 nm) [1]. Exosome biogenesis requires the processes of assembly and secretion. Exosomes may contain diverse cargos, such as proteins, lipids, and nucleic acids (DNA, mRNAs, and noncoding RNAs) [2,3,4]. Exosomal noncoding RNAs play a crucial role in regulating carcinogenesis [5]. Exosomal noncoding RNAs contain different types of nucleic acids, such as circular RNAs, long noncoding RNAs, and microRNAs (miRNAs). Literature reports indicate that cancer patients generally exhibit higher miRNAs in exosomes than normal controls [6]. Consequently, this review focuses on exosomal miRNAs.

Ferroptosis comprises iron-dependent non-apoptotic cell death characterized by the overexpression of membrane lipid peroxidation, an increase in cellular iron uptake, and the triggering of ferroptosis signaling [7]. Modulating ferroptosis is an anticancer strategy [8,9]. Ferroptosis-inducing compounds may improve pharmaceutical effects with respect to inhibiting metastasis, drug resistance, and tumor regression [9].

Notably, exosome and ferroptosis may interplay with each other [10,11,12,13,14,15]. Exosomes may enhance or suppress ferroptosis in several diseases and cancers [16]. The inhibition of ferroptosis may be triggered via exosome-mediated regulation that acts on ferroptosis signaling [10]. Exosomes may deliver biomolecules to recipient cells and, in turn, regulate ferroptosis-modulating signaling and response [11,12]. For example, breast cancerous exosomes suppress migration mediated by ferroptosis [13]. Bladder-cancer-isolated exosomes demonstrate ferroptosis-inducing effects by transporting miR-217 [14]. Exosomes isolated from the adipose tissue macrophages of an obesity-induced cardiac injury trigger ferroptosis by targeting ferroptosis-modulating signaling [17]. Consequently, drugs that regulate ferroptosis may modulate cancer and other medical therapies. Although exosome and ferroptosis exhibit an interplay relationship, this review only focuses on exploring the impact of ferroptosis on exosome biogenesis regarding the ferroptosis-modulating natural products and miRNAs.

### 1.2. Exosome-Biogenesis-Modulating Genes

**Figure 1:**
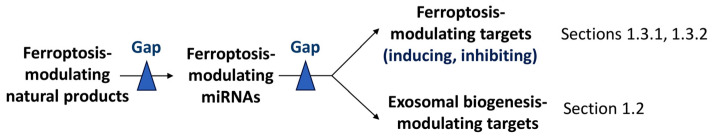
The rationale of ferroptosis-modulating miRNAs of ferroptosis-modulating natural products and their potential targeting of ferroptosis and exosome biogenesis modulation. There are two knowledge gaps for the natural product–miRNA–ferroptosis/exosome biogenesis target axis. The first knowledge gap is the disconnection between the modulating effects of miRNAs and natural products acting on ferroptosis. The second knowledge gap is disconnection between the ferroptosis- and exosome-biogenesis-modulating targets and natural-product-regulated miRNAs. This review focuses on retrieving natural products with ferroptosis-modulating effects. The involvement of miRNAs in ferroptosis-modulating natural products is explored by Google Scholar to fill the first gap. To fill the second gap, these ferroptosis-modulating miRNAs are used to retrieve the potential targets for ferroptosis and exosome biogenesis by utilizing Google Scholar and the miRDB database. Notably, the modulations of ferroptosis and exosome biogenesis are based on miRDB retrieval to identify the possible targets for ferroptosis and exosome biogenesis by these ferroptosis-modulated miRNAs. Ferroptosis-inducing genes, ferroptosis-inhibiting genes, and exosome-biogenesis-modulating genes are mentioned (Section 1.2, Section 1.3.1 and Section 1.3.2). Consequently, the rationale for the natural product–miRNA–ferroptosis/exosome biogenesis target axis is established.

The search methodology for exosome biogenesis genes was performed bioinformatically via the Gene Oncology database [18]. Exosome biogenesis genes such as secretion and assembly were summarized from the Gene Oncology (GO:1990182) in Mouse Genome Database [18] (accessed on 1 March 2023), including ATPase class II, type 9A (*ATP9A*) and *ATP13A2*; CD34 antigen (*CD34*); charged multivesicular body protein 2A (*CHMP2A*); COP9 signalosome subunit 5 (*COPS5*); hepatocyte growth-factor-regulated tyrosine kinase substrate (*HGS*); myosin VB (*MYO5B*); parkin RBR E3 ubiquitin protein ligase (*PRKN*); programmed cell death 6 interacting protein (*PDCD6IP*); RAB11A, a member of the RAS oncogene family (*RAB11A*), *RAB7A*, *RAB7B*, and *RAB27A*; syndecan 1 (*SCD1*) and *SDC4*; syndecan-binding protein (*SDCBP*); sphingomyelin phosphodiesterase 3; neutral (*SMPD3*); SNF8 subunit of the endosomal sorting complexes required for transport (ESCRT)-II complex (*SNF8*); SH3 domain and ITAM motif (*STAM*); STEAP family member 3 (*STEAP3*); tumor susceptibility gene 101 (*TSG101*); vacuolar protein sorting 4A (*VPS4A*); and *VPS4B*. These exosomal biogenesis-modulating genes (Figure 1) are used as candidates for determining the potential regulation of exosome biogenesis by carrying out a literature search (PubMed and Google Scholar) and bioinformatic data-mining (miRDB) [19]. These exosome-biogenesis-modulating genes are the potential target candidates for natural-product-regulated miRNAs, but they still need further assessment.

### 1.3. Ferroptosis-Modulating Genes

Several ferroptosis-modulating genes, such as 25 ferroptosis-inducing genes and 24 ferroptosis-inhibiting genes, are collected in this review. These ferroptosis-modulating genes (Figure 1) are used as candidates for determining the potential regulation of ferroptosis by carrying out a literature search (PubMed and Google Scholar) and bioinformatic data-mining (miRDB) [19].

#### 1.3.1. Ferroptosis-Inducing Genes

As stated above, twenty-five ferroptosis-inducing genes (Figure 1) were reported [20,21,22]. These include the following: Acyl-CoA synthetase long-chain family member 4 (*ACSL4*); activating transcription factor 3 (*ATF3*) and *ATF4*; autophagy-related 5 (*ATG5*) and *ATG7*; arachidonate 12-lipoxygenase (*ALOX12*), *ALOX15*, *ALOX5*, and *ALOXE3*; dipeptidyl-peptidase 4 (*DPP4*); endothelial PAS domain protein 1 (*EPAS1*; *HIF2A*); heme oxygenase-1 (*HMOX1*; *HO-1*); iron-responsive element-binding protein 2 (*IREB2*); lysophosphatidylcholine acyltransferase 3 (*LPCAT3*); microtubule-associated protein 1 light chain 3 alpha (*MAP1LC3A*); microtubule-associated protein 1 light chain 3 beta (*MAP1LC3B*); nuclear receptor coactivator 4 (*NCOA4*); cytochrome p450 oxidoreductase (*POR*); spermidine N1-acetyltransferase 1 (*SAT1*); transferrin (*TF*); transferrin receptor (*TFRC*); voltage-dependent anion channel 2 (*VDAC2*) and *VDAC3*; Yes 1-associated transcriptional regulator (*YAP1*); and WW domain-containing transcription regulator 1 (*WWTR1*).

#### 1.3.2. Ferroptosis-Inhibiting Genes

Twenty-four ferroptosis-inhibiting genes (Figure 1) were reported [20,23,24,25,26,27,28,29]. These include apoptosis-inducing factor mitochondria-associated 1 (*AIFM1*) and *AIFM2*; biglycan (*BGN*); ceruloplasmin (*CP*); ferritin heavy chain 1 (*FTH1*); ferritin light chain (*FTL*); ferritin mitochondrial (*FTMT*); glutamate–cysteine ligase catalytic subunit (*GCLC*); glutathione synthetase (*GSS*); glutathione peroxidase 4 (*GPX4*); hypoxia-inducible factor 1 subunit alpha (*HIF1A*); NEDD4 E3 ubiquitin protein ligase (*NEDD4*); nuclear factor erythroid 2 like 2 (*NFE2L2*; *NRF2*); prominin 2 (*PROM2*); solute carrier family 3 member 2 (*SLC3A2*); solute carrier family 7 member 11 (*SLC7A11*; *xCT*); solute carrier family 40 member 1 (*SLC40A1; FPN*); solute carrier family 11 member 2 (*SLC11A2*; *DMT1*); SP1 transcription factor (*SP1*); transcription factor AP-2 gamma (*TFAP2C*); tumor protein p53 (*TP53*); thioredoxin (*TXN*); transducin beta-like 1X-linked (*TBLR1*); and thioredoxin reductase 1 (*TXNRD1*). These ferroptosis-modulating genes are the potential target candidates for natural-product-regulated miRNAs, but still need further evaluation.

### 1.4. The Knowledge Gaps of miRNA-Modulating Natural Products for the Induction and Inhibition of Ferroptosis and Exosome Biogenesis

Several natural products were evaluated, and they demonstrated the modulation function for exosomal miRNAs and exosome biogenesis [30]. However, this exosomal miRNA review did not consider the impact of ferroptosis in natural product studies.

Several literature reports have recently organized comprehensive ferroptosis-modulating natural products [31,32,33,34]. For example, the effects of various natural products exhibiting inducing and inhibiting effects on ferroptosis in several diseases and cancers have been summarized [31,32]. Their functional targets and experimental models for these ferroptosis-modulating natural products are demonstrated. In comparison, the chemical structures of ferroptosis-modulating natural products are presented but do not discuss the target genes of ferroptosis [32,34]. Another review provides information on the compound source, cell line names, concentration, and treatment time of natural products with ferroptosis, necroptosis, and pyroptosis-inducing ability [33]. However, these reviews summarize ferroptosis-modulating natural products without considering the impact of miRNAs [31,32,33,34].

Some studies focused on exploring the role of miRNAs in regulating ferroptosis [35,36,37]. Several miRNAs that modulate cardiomyopathy, neuronal injury, and cancer-associated ferroptosis are summarized [35]. Ferroptosis-regulating miRNAs [36,37] and circular RNAs [36] occurring in several cancer cells are summarized [36]. The cell line models and targets for these ferroptosis-modulating miRNAs are provided. However, these reported miRNA functions focus on regulating ferroptosis without considering the contribution of natural products [35,36,37].

As mentioned, those studies of ferroptosis-modulating natural products and miRNAs were individually investigated without exploring their relationships. A knowledge gap was discovered in the connection between the modulating effects of miRNAs and natural products acting on ferroptosis. Moreover, there is another knowledge gap: the ferroptosis- and exosome-biogenesis-modulating targets of natural products-regulated miRNAs were limitedly reported. Hence, comprehensive assessments of natural product studies targeting the modulation of ferroptosis and exosome biogenesis by miRNAs are warranted.

### 1.5. The Novelty, Rationale, and Outline of This Review

Several reviews have specific scopes for natural products, miRNAs, exosome biosynthesis, or ferroptosis, but the connection between them is weakly emphasized and organized. The novelty of the current review is to fix these disconnections by introducing bioinformatics retrieval and deep literature searches.

Many types of cell death play differential roles in anticancer responses [38,39]. Interestingly, ferroptosis comprises iron-dependent non-apoptotic cell death. Switching from apoptosis to ferroptosis may improve the anticancer effects against cancer stem cells [40]. Moreover, many natural products exhibit modulating effects on ferroptosis. Therefore, this review focuses on ferroptosis-associated responses involving natural products. The rationale of this review is to propose a regulatory axis where ferroptosis-modulating natural products affect ferroptosis-modulating miRNAs, which in turn control ferroptosis- and exosome-biogenesis-modulating targets (Figure 1). Those two gaps are innovatively filled in this review as described below.

In this review, several ferroptosis-modulating natural products and their potential role of modulating ferroptosis signaling were overviewed by a literature search (PubMed and Google Scholar) (Section 2). There is a knowledge gap with respect to the connections between the modulating effects of the miRNA of natural products and ferroptosis and exosome biogenesis (Figure 1). To fill this gap, we performed a literature search (PubMed and Google Scholar) and bioinformatic database mining (miRDB) that allowed ferroptosis-modulating miRNAs and their potential ferroptosis-targeting genes to be retrieved (Section 3). Similarly, ferroptosis-modulating miRNAs and their potential exosome biogenesis-targeting genes were retrieved (Section 4).

Another gap is the connection between ferroptosis-modulating natural products and miRNAs because they were individually reported (Figure 1). After the literature search, the relationship between ferroptosis-modulating miRNAs and some natural products was explored to fill this gap (Section 5). Consequently, this review provides the connecting information between the potential modulation of miRNAs and exosome biogenesis in ferroptosis-modulating natural products.

## 2. Ferroptosis-Modulating Natural Products

Many natural products showing ferroptosis-modulating effects are well-reviewed [31,32,33,34]. Generally, these studies provide one or two ferroptosis-modulating targets from literature reports. However, these reports did not assess most of the 49 ferroptosis-modulating genes mentioned (Figure 1). Three main concerns are described as follows: (1) Ferroptosis-inducing (Section 2.1) and (2) ferroptosis-inhibiting (Section 2.2) natural products and their ferroptosis-modulating targets are illustrated. Finally, (3) the regulation of exosome biogenesis by ferroptosis-modulating natural products is discussed (Section 2.3).

The Google Scholar search methodology for ferroptosis-modulating natural products was performed as follows: Based on the search term “natural products ferroptosis”, only pure compounds are included for natural products with ferroptosis-modulating effects (inducing and inhibiting) (Table 1), whereas the crude extracts are excluded. Moreover, the ferroptosis-inducing and inhibiting genes (as described in Figure 1 or Section 1.3.1 and Section 1.3.2) regulated by these natural products are also retrieved by Google Scholar.

**Table 1:** Ferroptosis-modulating natural products and their responses to ferroptosis-inducing and ferroptosis-inhibiting genes.

	Natural Products	Ferroptosis Modulation by Natural Products *	Ferroptosis-Inducing Genes	Ferroptosis-Inhibiting Genes
Ferroptosis inducers	Artesunate [41]	Lymphoma	↑ ATG5 [42], ATG7 [43], NCOA4 [44], TFRC [45]	↓ SLC11A2 [46], FTH1 [47], GPX4 [48], SP1 [49]
	Albiziabioside A [50]	Breast Ca		↓ GPX4 [51]
	Alloimperatorin [24]	Breast Ca		↓ SLC7A11, GPX4, p-AIFM1 [24]
	Amentoflavone [52]	Gastric Ca		↓ FTH1 [31], SP1 [53]
	Ardisiacrispin B [54]	Leukemia		
	Aridanin [55]	Liver Ca		
	Artemisinin [56]	Osteosarcoma	↑ ATF3 [57]	↓ GPX4 [58]
	Artenimol [59]	Leukemia		
	Auriculasin [25]	Colon Ca		↓ pAIFM1 [25]
	Bromelain [60]	Colon Ca	↑ ATG5, ATG7 [61], ACSL4 [60]	
	Curcumin [62]	Colon Ca	↑ ATF3 [63], ACSL4 [64], IREB2 [63], HMOX1 [65]	↓ SLC7A11, GPX4 [62,64], HIF1A [66], NEDD4 [67,68]
	Dihydroartemisinin [69]	Glioma	↑ ATF4 [70,71], NCOA4 [72], HMOX1 [69]	↓ GPX4, SLC7A11, SLC3A2 [70], FTH1 [73]
	Dihydroisotanshinone I [74]	Glioma	↑ ACSL4 [74]	↓ GPX4 [74]
	Diplacone [75]	Lung Ca		
	DMOCPTL [76]	Breast Ca		↓ GPX4 [76]
	Epigallocatechin gallate [77]	Pancreatic Ca	↑ ATF4 [78], ATG5, ATG7 [79], YAP1 [80]	↓ SP1, TP53 [81]
	Epunctanone [82]	Leukemia		
	Erianin [83]	Renal Ca stem cells	↑ ALOX12 [83]	↓ GPX4, FTH1, SLC7A11 [83], TP53 [84]
	Ferroptocide [23]	Ovarian Ca		↓ TXN [23]
	Gallic acid [85]	Breast Ca	↑ ATF4 [86]	↓ GPX4, SLC7A11 [86]
	Heteronemin [87]	Liver Ca	↑ ATG5, ATG7 [88]	↓ GPX4 [87], TP53 [89,90]
	Matrine [91]	Colon Ca	↑ ATF4 [91]	↓ GPX4, SLC7A11 [91]
	Nitidine chloride [92]	Myeloma		↓ NEDD4 [93]
	Piperlongumine [94]	Pancreas Ca	↑ ATF4 [95], HMOX1 [96]	↓ FTH1, SLC7A11, GPX4 [97], SP1 [98]
	Pseudolaric acid B [99]	GBM	↑ TFRC [31]	↓ SLC7A11 [99]
	Punicic acid [100]	Colon Ca		
	Quercetin [101]	Breast Ca	↑ ATF3 [102], HMOX1 [103]	↓ SLC40A1 [104], FTL [105], SP1 [106]
	Ruscogenin [31]	Pancreas Ca	↑ TF [31,107]	↓ SLC40A1 [107]
	Salinomycin [108]	Head/neck Ca	↑ ATF3 [109], ATF4 [110], ATG5, ATG7 [111], DPP4 [112], IREB2, TFRC [108,113]	↓ FTH1, FTL [113], NFE2L2, GCLC [114], HIF1A [115], TP53 [116], FTH1 [108]
	Sanguinarine [117,118]	Cervical Ca		↓ SLC7A11 [118]
	Solasonine [31]	Liver Ca		↓ GPX4, GSS [119], SLC7A11 [120]
	Sulforaphane [121]	Leukemia	↑ ATF3 [122], ATG7 [123], ALOX12 [124], HMOX1 [125], YAP1 [126]	↓ GPX4 [124], SLC7A11 [127]
	Tagitinin C [128]	Colon Ca	↑ HMOX1 [128]	
	Talaroconvolutin A [22]	Colon Ca	↑ ALOXE3 [22]	↓ SLC7A11 [22]
	Trigonelline [31]	Liver Ca	↑ DPP4 [129]	↓ NFE2L2 [129]
	Typhaneoside [130]	Leukemia		
	Ungeremine [131]	Leukemia		
	Withaferin A [31]	Neuroblastoma	↑ ATF3, ATF4, HMOX1 [132], ATG5, ATG7 [133]	↓ GPX4, NFE2L2 [31]
	β-Elemene [134]	Colon Ca		↓ HIF1A [135], SP1 [136]
	β-Phenethyl isothiocyanate (PEITC) [137]	Osteosarcoma	↑ ATG4 [138], HMOX1 [139]	↓ SLC11A2, SLC40A1, FTH1 [140], GPX4, SLC11A2 [137], HIF1A [141]
Ferroptosis inhibitors	Nodosin [142]	Bladder Ca		↑ AIFM2, GPX4 [142]
	Nordihydroguaiareticacid [143]	Leukemia	↓ ALOX12, ALOX15 [143]	
	Cryptotanshinone [144]	Pancreas Ca		
	Artepillin C [145]	Neuron		
	Bakuchiol [146]	Neuron		
	Berberine [147]	Cardiomyocytes	↓ ATG5 [148], ACSL4 [149]	↑ Cruloplasmin [150], NFE2L2 [151], SLC7A11 [149]
	Glycyrrhizin [152]	Acute liver failure		↑ GPX4 [153], SLC40A1 [154]
	Psoralidin [146]	Neuron	↓ ALOX5 [146]	
	Butein [155]	BMSCs		↑ SP1 [156]
	Baicalein [157]	Pancreas Ca	↓ ATF3 [158], ACSL4 [157], ALOX12 [159], ALOX15 [160]	↑ GPX4 [157,161], SLC7A11 [157], GCLC [162]
	7-O-cinnamoyl-taxifolin [163]	Neuron		↑ NFE2L2 [163]
	3-Hydroxybakuchiol [146]	Neuron		
	Morachalcone D [164]	Cardiomyocytes		↑ SLC7A11, NFE2L2, GPX4 [164]
	Sterubin [165]	Neuron		↑ NFE2L2 [165]
	Proanthocyanidin [166]	Spinal cord injury mice	↓ ATG5, ATG7 [167], ACSL4 [168], DDP4 [169]	↑ GPX4, SLC7A11 [168], NFE2L2 [151]
	Puerarin [170]	Cardiomyocyte injury		↑ FTH1 [31], GCLC [171], NFE2L2, GPX4 [172]

* All ferroptosis-inducing natural products show antiproliferative effects on cancer cells, while ferroptosis-inhibiting natural products show protective effects on ferroptosis-induced cell death of cancer cells and non-cancer cell injury. Ca, cancer; GBM, glioblastoma multiforme. Ferroptosis-inducing (top) and ferroptosis-inhibiting (bottom) genes have been mentioned (Section 1.3). The blank column indicates data are not available by Google Scholar retrieval. ↓ and ↑ indicate the inhibiting and inducing effects by natural products.

### 2.1. Ferroptosis-Inducing Natural Products

Several natural products are potential ferroptosis inducers (Table 1). Although the review focuses on ferroptosis, some ferroptosis-inducing natural products with ferroptotic and non-ferroptotic effects are mentioned. Some reviews on ferroptosis-inducing natural products did not examine ferroptosis but showed the impact of ferroptosis by the modulation of ferroptosis-inducing or ferroptosis-inhibiting genes.

**Figure 2:**
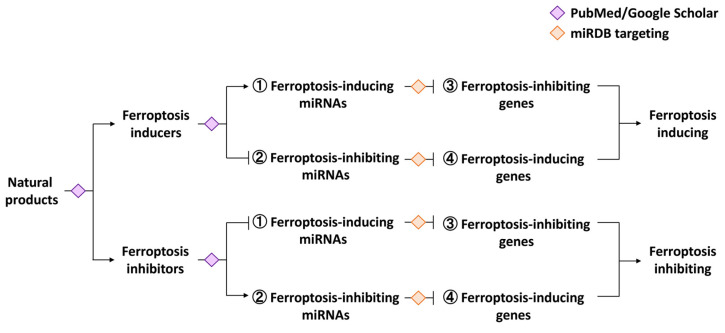
Connecting ferroptosis-modulating natural products to their regulating miRNAs and targets. Potential ferroptosis inducers may upregulate ferroptosis-inducing miRNAs and/or downregulate ferroptosis-inhibiting miRNAs and, in turn, suppress the functions of ferroptosis-inhibiting and/or ferroptosis-inducing genes (Section 2.1). Similarly, the potential ferroptosis inhibitors may upregulate ferroptosis-inhibiting miRNAs and/or downregulate ferroptosis-inducing miRNAs, which, in turn, suppress the functions of ferroptosis-inducing and/or ferroptosis-inhibiting genes (Section 2.2).

**Figure 3:**
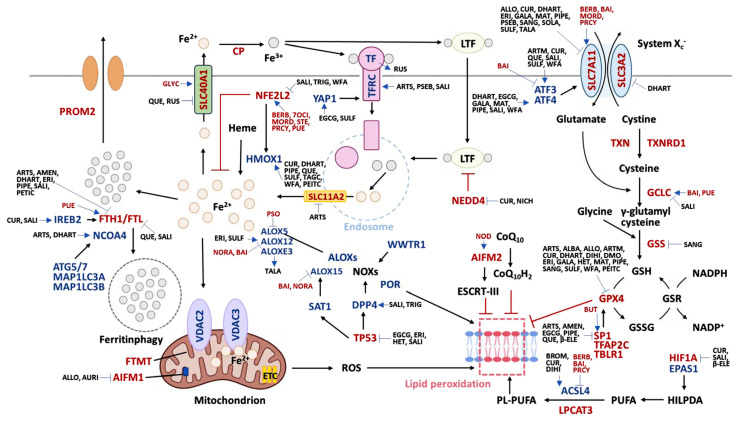
Natural products that modulate specific proteins in the molecular pathway of inducing and inhibiting ferroptosis. This pathway is drawn by considering the information from many literature reports [173,174,175,176,177,178,179,180,181,182,183,184,185,186,187,188,189]. Natural products that target these specific proteins have been described in Table 1. The ferroptosis-inducing and inhibiting natural products are indicated in black and red, respectively. Although these natural products potentially target the ferroptosis signaling pathway, their potential impact on ferroptosis still warrants detailed investigation. The ferroptosis-inducing and inhibiting targets (as described in Section 1.3.1 and Section 1.3.2) are indicated in blue and red, respectively. Natural products that may induce the targets are indicated by an arrow line, while natural products that may inhibit the targets are indicated by a “T” line. Abbreviations (natural products): Albiziabioside A (ALBA), Alloimperatorin (ALLO), Amentoflavone (AMEN), Artemisinin (ARTM), Artesunate (ARTS), Auriculasin (AURI), Baicalein (BAI), Berberine (BERB), Bromelain (BROM), Butein (BUT), Curcumin (CUR), Dihydroartemisinin (DHART), Dihydroisotanshinone I (DIHI), DMOCPTL (DMO), Epigallocatechin Gallate (EGCG), β-Elemene (βELE), Erianin (ERI), Gallic acid (GALA), Glycyrrhizin (GLYC), Heteronemin (HET), Matrine (MAT), Morachalcone D (MORD), Nitidine chloride (NICH), Nodosin (NOD), Nordihydroguaiaretic acid (NORA), 7-O-cinnamoyl-taxifolin (7OCI), β-Phenethyl isothiocyanate (PEITC), Piperlongumine (PIPE), Proanthocyanidin (PRCY), Pseudolaric acid B (PSEB), Psoralidin (PSO), Puerarin (PUE), Quercetin (QUE), Ruscogenin (RUS), Salinomycin (SALI), Sanguinarine (SANG), Sulforaphane (SULF), Solasonine (SOLA), Sterubin (STE), Talaroconvolutin A (TALA), Trigonelline (TRIG), Tagitinin C (TAGC), Withaferin A (WFA).

Generally, natural products upregulating the ferroptosis-inducing genes or downregulating the ferroptosis-inhibiting genes are potential ferroptosis inducers (Figure 2). The ferroptosis-inducing natural products and miRNAs were retrieved by a literature search (PubMed and Google Scholar), while potential targets of ferroptosis-inducing RNAs were retrieved from the miRDB database [19]. The ferroptosis-inducing functions of the reported natural products and their modulations (inducing or inhibiting) on ferroptosis-inducing or ferroptosis-inhibiting targets are exemplified as follows (Table 1). Natural products that modulate specific proteins in the molecular pathway of inducing ferroptosis are shown (Figure 3).

#### 2.1.1. Artesunate

Several artesunate studies demonstrate the upregulation of ferroptosis-inducing genes (Table 1). Artesunate preferentially inhibits the proliferation of head and neck cancer cells with low cytotoxicity relative to normal cells by promoting ferroptosis [190]. Artesunate causes ferritinophagy, i.e., ferritin (FTH1 and FTL)-degradation-dependent ferroptosis, by upregulating the ferroptosis-inducing *NCOA4* gene and downregulating ferroptosis-inhibiting genes (*FTH1* and *FTL*) [44]. In addition to ferroptosis, artesunate triggers endoplasmic reticulum (ER) stress in Burkitt’s lymphoma cells, accompanied by upregulating the ferroptosis-inducing *ATF4* gene [41].

Without assessing ferroptosis, artesunate induces apoptosis [42], autophagy [43], and anti-angiogenesis [45] effects by upregulating ferroptosis-inducing genes (Table 1). Artesunate induces the antiproliferation and apoptosis of endometrial cancer cells, accompanied by upregulating the ferroptosis-inducing *ATG5* gene, which enhances the cytotoxicity of natural killer cells. In contrast, the knockdown of *ATG5* reduces the cytotoxicity of natural killer cells [42]. *ATG7* knockdown inhibits the artesunate-induced autophagy of cervical cancer cells [43], suggesting that artesunate may upregulate the ferroptosis-inducing *ATG7* gene. Moreover, artesunate inhibits renal cancer cell proliferation, migration, and angiogenesis by upregulating the ferroptosis-inducing *TFRC* gene [45].

In contrast, several artesunate studies demonstrate the downregulation of ferroptosis-inhibiting genes associated with ferroptosis and/or other non-ferroptosis responses (Table 1). Artesunate alleviates ocular fibrosis by promoting ferroptosis associated with downregulating the ferroptosis-inhibiting *GPX4* gene [48]. Artesunate triggers the apoptosis of leukemia cells by downregulating the ferroptosis-inhibiting *SLC11A2* gene [46]. Artesunate improves the antiproliferation and apoptosis of lymphoblasts [46] by downregulating the ferroptosis-inhibiting *SLC40A1* gene [46]. Artesunate enhances anti-human cytomegalovirus (HCMV) effects by downregulating the ferroptosis-inhibiting *SP1* gene [49].

Artesunate may demonstrate both the upregulation of ferroptosis-inducing genes and the downregulation of ferroptosis-inhibiting genes associated with ferroptosis and/or other non-ferroptosis responses (Table 1). Artesunate induces ferritinophagy-mediated ferroptosis and the anti-fibrosis effects of activated hepatic stellate cells by upregulating the ferroptosis-inducing *ATG5* gene and downregulating ferroptosis-inhibiting *FTH1* gene [47]. Notably, the participation of ferroptosis in these natural products still warrants further assessment.

#### 2.1.2. Albiziabioside A and Alloimperatorin

The responses of the upregulation of ferroptosis-inducing genes and/or the downregulation of ferroptosis-inhibiting genes were also reported in several ferroptosis-inducing natural products, such as albiziabioside A and alloimperatorin (Table 1). Albiziabioside A triggers the ferroptosis of colon cancer cells, which is associated with apoptosis [50]. Similarly, albiziabioside A induces the apoptosis and ferroptosis of breast cancer cells, accompanied by the downregulation of the ferroptosis-inhibiting *GPX4* gene [51]. For alloimperatorin, it induces the antiproliferation, apoptosis, and ferroptosis of breast cancer cells by downregulating the ferroptosis-inhibiting genes (*SLC7A11*, *GPX4*, and phosphorylated AIFM1) [24]. The detailed impacts of these natural products on ferroptosis still warrant further assessment.

#### 2.1.3. Amentoflavone, Artemisinin, Auriculasin, and Bromelain

Similar ferroptosis modulation is also shown for amentoflavone, artemisinin, auriculasin, and bromelain (Table 1). Amentoflavone inhibits proliferation and triggers the autophagy-mediated ferroptosis of human gastric cancer cells, which is accompanied by downregulating the ferropto-sis-inhibiting *FTH1* gene [31]. Amentoflavone causes apoptosis in glioma cells by downregulating the ferroptosis-inhibiting *SP1* gene [53]. Artemisinin triggers the ferroptosis of osteosarcoma cells [56]. Artemisinin inhibits breast and lung cancer cell migration by upregulating the ferroptosis-inducing *ATF3* gene [57]. In comparison, artemisinin enhances the ferroptosis of cancer cells by downregulating the ferroptosis-inhibiting *GPX4* gene [58]. Auriculasin, a *Flemingia philippinensis*-derived flavonoid, triggers the ferroptosis and apoptosis of colon cancer cells by downregulating the ferroptosis-inhibiting protein, such as phosphorylated AIFM1 [25]. For bromelain, it suppresses hepatic lipid accumulation by upregulating ferroptosis-inducing proteins, such as phosphorylated ATG5 and ATG7, in the liver of high-fat-diet-fed mice [61]. In comparison, bromelain inhibits proliferation and induces the ferroptosis of colon cancer cells by upregulating the ferroptosis-inducing *ACSL4* gene [60]. The impacts on the ferroptosis of these natural products still warrant further assessment.

#### 2.1.4. Curcumin

In the case of the upregulation of ferroptosis-inducing genes, several curcumin studies demonstrate the induction of ferroptosis (Table 1). Curcumin enhances the ferroptosis of lung cancer cells by upregulating the ferroptosis-inducing *ACSL4* gene [64]. Curcumin promotes ferroptosis, thereby inhibiting lung cancer tumor growth, by upregulating *ACSL4* and downregulating ferroptosis-inhibiting genes (*SLC7A11* and *GPX4*) [64]. In addition to ferroptosis, curcumin promotes the autophagy of lung cancer cells, and this is alleviated by downregulating the ferroptosis-inhibiting *IREB2* gene [63]. This suggests that curcumin-induced ferroptosis and autophagy are associated with the upregulation of *IREB2*.

Without assessing ferroptosis, curcumin induces apoptosis [63,65] by upregulating ferroptosis-inducing genes (Table 1). Curcumin causes the antiproliferation and apoptosis of leiomyoma cells by inducing the expression of the ferroptosis-inducing *ATF3* gene [63]. Similarly, curcumin triggers the apoptosis of breast cancer cells by promoting the expression of the ferroptosis-inducing *HMOX1* gene [65].

In the case of the downregulation of ferroptosis-inhibiting genes, several curcumin studies demonstrate the induction of ferroptosis (Table 1). Curcumin promotes the antiproliferation and ferroptosis of the colon [62] and lung [64] cancer cells by downregulating ferroptosis-inhibiting genes (*SLC7A11* and *GPX4*). Without assessing ferroptosis, curcumin modulates non-ferroptosis effects such as tumor neovascularization [66] and migration [67,68] by downregulating ferroptosis-inhibiting genes (Table 1). Curcumin inhibits the gene expression of tumor neovascularization, such as that of the ferroptosis-inhibiting *HIF1A* gene, in pituitary adenomas [66]. Curcumin suppresses the proliferation and migration of prostate [68] and glioma [67] cancer cells by inhibiting the expression of the ferroptosis-inhibiting *NEDD4* gene.

#### 2.1.5. Dihydroartemisinin, Dihydroisotanshinone I, and DMOCPTL

The upregulation of ferroptosis-inducing genes and/or the downregulation of ferroptosis-inhibiting genes were demonstrated in several ferroptosis-inducing natural products, such as dihydroartemisinin, dihydroisotanshinone I, and DMOCPTL (Table 1).

Dihydroartemisinin, a common artemisinin derivative, triggers the ferroptosis of several cancer and non-cancer cells (Table 1). In cancer cell studies, dihydroartemisinin inhibits proliferation and migration and triggers the ferroptosis of glioma cells by upregulating the ferroptosis-inducing *HMOX1* gene and downregulating the ferroptosis-inhibiting *GPX4* gene [69]. Dihydroartemisinin promotes the antiproliferation and ferroptosis of liver cancer cells by upregulating the ferroptosis-inducing *ATF4* gene and downregulating ferroptosis-inhibiting genes (*GPX4*, *SLC7A11*, and *SLC3A2*) [70]. In non-cancer cell studies, dihydroartemisinin triggers the ferroptosis of hepatic stellate cells by upregulating the ferroptosis-inducing *NCOA4* gene [72]. Moreover, dihydroartemisinin also induces a non-ferroptosis response, such as ER stress. Dihydroartemisinin also induces the ER stress of porcine ovarian granulosa cells by upregulating the ferroptosis-inducing *ATF4* gene [71]. However, its impact on the regulation of ferroptosis warrants a detailed assessment.

Dihydroisotanshinone I suppresses proliferation and induces the ferroptosis of glioma cells by upregulating the ferroptosis-inducing *ACSL4* gene and downregulating the ferroptosis-inhibiting *GPX4* gene [74] (Table 1). Moreover, DMOCPTL, a derivative of the natural product parthenolide, induces the apoptosis and ferroptosis of breast cancer cells by directly binding to GPX4 and causing degradation of GPX4 [76]. The impacts on ferroptosis of these natural products still warrant further investigation.

#### 2.1.6. Epigallocatechin Gallate (EGCG)

Similar ferroptosis modulation is attributed to EGCG (Table 1). EGCG may upregulate and downregulate ferroptosis-inducing and inhibiting genes, respectively. For the modulation of ferroptosis-inducing genes, EGCG enhances the ferroptosis of pancreatic cancer cells by promoting the degradation of the ferroptosis-inhibiting *GPX4* gene [77]. For non-ferroptosis responses, EGCG may induce ER stress [78], autophagy [79,80], and apoptosis [79]. EGCG triggers the ER stress of colon cancer cells by upregulating the ferroptosis-inducing *ATF4* gene [78]. EGCG induces the autophagy-mediated death of breast cancer cells by retaining the ferroptosis-inducing *YAP1* gene in the cytoplasm [80]. EGCG induces the antiproliferation, apoptosis, and autophagy of umbilical vein endothelial cells grown on 316L stainless steel by upregulating ferroptosis-inducing genes (*ATG5* and *ATG7*) [79].

In contrast, EGCG may downregulate ferroptosis-inhibiting genes (Table 1). Without assessing ferroptosis, EGCG modulates non-ferroptosis effects such as apoptosis. EGCG suppresses proliferation and causes the apoptosis of oral cancer cells by downregulating the ferroptosis-inhibiting *TP53* gene [81]. Moreover, EGCG also downregulates the ferroptosis-inhibiting *SP1* gene in terms of molecular docking experiments [81]. The ferroptosis response of these non-ferroptosis studies of EGCG warrants a detailed investigation.

#### 2.1.7. Erianin and Ferroptocide

Similar ferroptosis modulation is attributed to erianin and ferroptocide (Table 1). Erianin induces the ferroptosis of renal cancer stem cells by upregulating the ferroptosis-inducing *ALOX12* gene [83] and downregulating ferroptosis-inhibiting genes (*GPX4*, *FTH1*, and *SLC7A11*) [83]. Erianin causes the antiproliferation of cervical cancer cells by downregulating the ferroptosis-inhibiting *TP53* gene [84], but the impact of ferroptosis warrants a detailed investigation.

Ferroptocide, a novel compound derived from pleuromutilin, promotes the ferroptosis of ovarian cancer cells [23] (Table 1). Ferroptocide is also a TXN inhibitor [23] with the potential to inhibit antioxidant systems and cause oxidative stress, but its ferroptotic effects warrant a detailed assessment.

#### 2.1.8. Gallic Acid, Heteronemin, Matrine, Nitidine Chloride, and Sanguinarine

By regulating ferroptosis-modulating genes, several studies using gallic acid, heteronemin, matrine, nitidine chloride, and sanguinarine are exemplified (Table 1). Gallic acid induces the ferroptosis of breast cancer cells [85]. In addition to ferroptosis, gallic acid also triggers the apoptosis of breast cancer cells by downregulating the ferroptosis-inhibiting *GPX4* gene [85]. Similarly, gallic acid suppresses the proliferation of colon cancer cells, which is reversed by a ferroptosis inhibitor [86], indicating that gallic acid is a ferroptosis inducer. Mechanistically, gallic acid regulates the ferroptosis of colon cancer cells by upregulating the ferroptosis-inducing *ATF4* gene and downregulating ferroptosis-inhibiting genes (*GPX4* and *SLC7A11*) [86].

Heteronemin, a marine terpenoid, triggers the apoptosis and ferroptosis of liver cancer cells by downregulating *GPX4* [87] (Table 1). Heteronemin also regulates several ferroptosis modulators in cancers. In the case of pancreatic cancer cells, heteronemin causes ferroptosis-based cell death by upregulating ferroptosis-inducing genes (*ATG5* and *ATG7*) [88]. Without assessing ferroptosis, heteronemin inhibits lung [89] and oral [90] cancer cell growth and downregulates the ferroptosis-inhibiting *TP53* gene. However, more of its ferroptotic effects warrant a detailed examination.

Literature reports support both matrine and nitidine chloride as potential ferroptosis inducers. Matrine shows antiproliferation and promotes the ferroptosis of colon cancer cells by upregulating the ferroptosis-inducing *ATF4* gene and downregulating the ferroptosis-inhibiting genes (*GPX4* and *SLC7A11*) [91]. Similarly, nitidine chloride, a natural product derived from the traditional Chinese medicine *Zanthoxylum nitidum*, induces ferroptosis in multiple myeloma [92]. A study mentioned that nitidine chloride inhibited the proliferation of lung cancer cells by downregulating the ferroptosis-inhibiting *NEDD4* gene [93]. Moreover, other natural benzophenanthridine alkaloids, such as sanguinarine [117], also induce ferroptosis in cervical cancer cells by downregulating *SLC7A11* [118].

#### 2.1.9. Piperlongumine and Pseudolaric Acid B

Similar ferroptosis modulation is attributed to piperlongumine and pseudolaric acid B (Table 1). Piperlongumine triggers the ferroptosis and cell death of pancreatic cancer cells [94]. Piperlongumine has been reported to show anticancer effects by modulating ferroptosis inducers and inhibitors, but the impact of ferroptosis was not assessed. For example, piperlongumine induces the apoptosis of liver cancer cells by upregulating the ferroptosis-inducing *ATF4* gene [95]. Piperlongumine promotes the apoptosis of pancreatic cancer cells by upregulating the ferroptosis-inducing *HMOX1* gene [96]. Piperlongumine shows the antiproliferation of oral cancer cells by downregulating ferroptosis-inhibiting genes (*FTH1*, *SLC7A11*, and *GPX4*) [97]. Piperlongumine downregulates the ferroptosis-inhibiting *SP1* gene in kidney cancer cells [98]. Since the expressions of these ferroptosis inducers and inhibitors have changed, a detailed evaluation of ferroptosis induction in these cancer studies treated with piperlongumine is warranted. Moreover, pseudolaric acid B induces the antiproliferation and ferroptosis of glioma cells by downregulating the ferroptosis-inhibiting *SLC7A11* gene [99].

#### 2.1.10. Quercetin

Several quercetin studies demonstrate the upregulation of ferroptosis-inducing genes (Table 1). Quercetin promotes the ferroptosis of breast cancer cells by improving the lysosomal degradation of ferritin (FTH1 and FTL), which are ferroptosis-inhibiting proteins [101]. Quercetin was reported to upregulate several ferroptosis-inducing genes, but the impact of ferroptosis was not assessed. For example, quercetin enhances macrophage M2 polarization by inducing the expression of the ferroptosis-inducing *ATF3* gene [102]. Quercetin promotes lipopolysaccharide (LPS)-influenced NO generation to alleviate the inflammatory responses of microglial cells by upregulating the ferroptosis-inducing *HMOX1* gene [103]. This warrants a detailed investigation of the ferroptosis responses of the above quercetin studies.

In contrast, quercetin was reported to downregulate ferroptosis-inhibiting genes (*SP1* [104], *SLC40A1* [104], and *FTL* [105]), but the impact of ferroptosis was not assessed. In a cancer study, quercetin causes the antiproliferation and apoptosis of malignant pleural mesothelioma by inhibiting the expression of the ferroptosis-inhibiting *SP1* gene [106]. In non-cancer studies, quercetin downregulates the ferroptosis-inhibiting *SLC40A1* gene of colon cancer cells and reduces intestinal iron absorption in rats [104]. Similarly, quercetin reduces alcohol-fed mice’s liver damage and iron levels by downregulating *FTL* [105]. In contrast, other ferroptosis events are rarely investigated in these studies [104,105]. An assessment of the impact of quercetin treatments on ferroptosis is warranted in these studies.

#### 2.1.11. Ruscogenin, Sulforaphane, and Solasonine

Similar ferroptosis modulation is attributed to ruscogenin, solasonine, and sulforaphane (Table 1). Ruscogenin inhibits proliferation and triggers the ferroptosis of pancreatic cancer cells by upregulating the ferroptosis-inducing *TF* gene and downregulating the ferroptosis-inhibiting *SLC40A1* gene [107]. Moreover, sulforaphane is a ferroptosis inducer for leukemia cell death by downregulating the ferroptosis-inhibiting *GPX4* gene [121].

Solasonine, a *Solanum melongena*-derived natural product, inhibits proliferation and promotes the ferroptosis of liver cancer cells by downregulating ferroptosis-inhibiting genes (*GPX4* and *GSS*) [119]. Without assessing ferroptosis, solasonine induces cancer cell proliferation by downregulating ferroptosis-inhibiting genes. Solasonine inhibits the proliferation of pancreatic cancer cells by downregulating the ferroptosis-inhibiting *SLC7A11* gene in a ubiquitination–degradation manner [120]. The impact of ferroptosis on this solasonine study needs further assessment.

#### 2.1.12. Salinomycin

Salinomycin studies showing similar ferroptosis modulation (upregulation and downregulation) are exemplified (Table 1). Salinomycin induces ferroptosis in head and neck cancer cells by upregulating the ferroptosis-inducing *TFRC* gene and downregulating *FTH1* [108]. Without assessing ferroptosis, salinomycin induces non-ferroptotic effects, such as migration [109], ER stress [110], autophagy [111], and apoptosis [112], by upregulating ferroptosis-inducing genes (Table 1). Salinomycin inhibits prostate cancer growth and migration by upregulating the ferroptosis-inducing *ATF3* gene [109]. Salinomycin induces ER stress in prostate cancer cells associated with the upregulation of the ferroptosis-inducing *ATF4* gene [110]. Salinomycin induces the autophagy and cell death of breast cancer cells by upregulating ferroptosis-inducing genes (*ATG5* and *ATG7*) [111]. Salinomycin inhibits proliferation, induces apoptosis, and upregulates the expression of the ferroptosis-inducing *DPP4* gene in colon cancer cells [112].

For comparison, without assessing ferroptosis, salinomycin induces non-ferroptotic effects, such as ER stress [114] and apoptosis [114,115,116], by downregulating ferroptosis-inhibiting genes (Table 1). Salinomycin triggers ER stress and the apoptosis of prostate cancer cells, and this is associated with the downregulation of ferroptosis-inhibiting genes (*NFE2L2* and *GCLC*) [114]. Salinomycin decreases the proliferation and promotes the apoptosis of endometrial cancer cells by downregulating the ferroptosis-inhibiting *HIF1A* gene [115]. Salinomycin induces the apoptosis of liver cancer cells by downregulating the ferroptosis-inhibiting *TP53* gene [116]. Additionally, salinomycin is reported to be a SLC11A2 inhibitor in cancer stem cells by upregulating iron homeostasis [191].

Moreover, salinomycin may upregulate and downregulate ferroptosis-modulating genes. Salinomycin causes the antiproliferation of cancer stem cells by upregulating the ferroptosis-inducing genes (*IREB2* and *TFRC*) and downregulating ferritin consisting of *FTH1* and *FTL* [113].

#### 2.1.13. Sulforaphane

Sulforaphane studies showing the ferroptosis modulation (upregulation and downregulation) are exemplified (Table 1). Sulforaphane inhibits the proliferation of colon cancer cells by upregulating the ferroptosis-inducing *ATF3* gene [122]. Sulforaphane demonstrates ferroptosis-mediated antiproliferation of lung cancer cells by downregulating the ferroptosis-inhibiting *SLC7A11* gene [127].

Without assessing ferroptosis, sulforaphane induces non-ferroptotic effects, such as autophagy [123] and apoptosis [123,124], by modulating ferroptosis-inducing or inhibiting genes (Table 1). Sulforaphane induces the autophagy and apoptosis of hepatoblastoma cells, associated with the upregulation of the ferroptosis-inducing *ATG7* gene [123]. Sulforaphane induces the apoptosis of cervical cancer cells by enhancing the ferroptosis-inducing *ALOX12* gene and inhibiting the expression of the ferroptosis-inhibiting *GPX4* gene [124].

Some sulforaphane reports also regulate ferroptosis-modulating genes without assessing ferroptosis responses. Sulforaphane inhibits the proliferation of head and neck cancer cells by upregulating the ferroptosis-inducing *HMOX1* gene [125]. Sulforaphane inhibits the proliferation of cancer stem cells by upregulating the ferroptosis-inducing *YAP1* gene [126]. The impact of ferroptosis on these solasonine studies needs further assessment.

#### 2.1.14. Tagitinin C, Talaroconvolutin A, Trigonelline, and Withaferin A

Similar ferroptosis modulation is applied to tagitinin C, talaroconvolutin A, trigonelline, and withaferin A (Table 1). Tagitinin C, a *Tithonia diversifolia*-derived sesquiterpene lactone, triggers the antiproliferation and ferroptosis of colon cancer cells by upregulating the *HMOX1* ferroptosis-inducing gene [128]. Talaroconvolutin A, a *Talaromyces purpureogenus*-derived natural product, inhibits proliferation and promotes the ferroptosis of colon cancer cells by upregulating the ferroptosis-inducing *ALOXE3* gene and downregulating *SLC7A11* [22].

Trigonelline inhibits the ferroptosis-inducing *NFE2L2* gene [192]. The upregulation of *NFE2L2* inhibits the ferroptosis of liver cancer cells [193]. It can be concluded, therefore, that trigonelline is a ferroptosis inducer [31]. Furthermore, *DPP4* inhibition triggers the senescence of endothelial cells by upregulating *NFE2L2* [129]. Trigonelline is a potential activator for the ferroptosis-inducing *DPP4* gene, but its ferroptotic effects warrant a detailed investigation.

Withaferin A is a ferroptosis inducer and tumor-suppressing agent of neuroblastoma [194]. Without assessing ferroptosis, withaferin A induces non-ferroptotic effects, such as apoptosis [132] and autophagy [133], by modulating ferroptosis-inducing genes (Table 1). Withaferin A induces the intrinsic apoptosis of glioblastoma cells by upregulating ferroptosis-inducing genes (*ATF3*, *ATF4*, and *HMOX1*) [132]. Withaferin A induces autophagy in liver-cancer-xenografted mice by upregulating ferroptosis-inducing genes (*ATG5* and *ATG7*) [133]. Ferroptosis responses within these withaferin A studies need further evaluation.

#### 2.1.15. β-Elemene and β-Phenethyl Isothiocyanate (PEITC)

Similar ferroptosis modulation is attributed to β-elemene and PEITC (Table 1). β-elemene demonstrating the downregulation of ferroptosis-inhibiting genes is exemplified. β-elemene induces the ferroptosis-mediated cell death of colon cancer cells [134]. Non-ferroptosis responses are also modulated by β-elemene. For example, β-elemene inhibits the proliferation of pancreatic cancer cells by reducing the expression of the ferroptosis-inhibiting *HIF1A* gene [135]. β-elemene inhibits the proliferation of lung cancer cells by downregulating the ferroptosis-inhibiting *SP1* gene [136].

In comparison, PEITC studies showing the downregulation of ferroptosis-inhibiting genes are exemplified. PEITC suppresses proliferation and promotes ferroptosis, apoptosis, and autophagy in osteosarcoma cells [137]. PEITC treatment triggers autophagy and the apoptosis of prostate cancer cells by upregulating the ferroptosis-inducing *ATG5* gene [138]. PEITC induces the expression of the ferroptosis-inducing *HMOX1* gene in primary mouse hepatocytes [139]. Moreover, PEITC induces ferroptosis, apoptosis, and autophagy by downregulating ferroptosis-inhibiting genes (*SLC11A2*, *SLC40A1*, and *FTH1*) in osteosarcoma [140]. Similarly, PEITC causes the ferroptosis, autophagy, and apoptosis of murine osteosarcoma cells by downregulating ferroptosis-inhibiting genes (*GPX4* and *SLC11A2*) [137]. PEITC downregulates the expression of the ferroptosis-inhibiting gene HIF1A in breast cancer cells [141]. The impact of ferroptosis on these PEITC studies needs further investigation.

#### 2.1.16. Other Ferroptosis-Inducing Natural Products

Some natural products (Table 1) also demonstrate ferroptosis-inducing effects, but their potential ferroptosis-modulating targets could not be retrieved by a literature search. For example, ardisiacrispin B induces the apoptosis and ferroptosis of breast cancer cells [54]. Aridanin [55], artenimol [59], and epunctanone [82] induce the antiproliferation and ferroptosis of leukemia cells, and this is reversed by ferroptosis-inhibiting genes. Diplacone, a *Paulownia tomentosa* fruit-derived natural product, causes the antiproliferation and ferroptosis of lung cancer cells [75]. Punicic acid, a main bioactive component in pomegranate seed oil, exhibits antiproliferation and triggers ferroptosis in colon cancer cells [100]. Typhaneoside inhibits the proliferation of acute myeloid leukemia (AML) by upregulating ferroptosis and autophagy [130]. Ungeremine inhibits the proliferation of leukemia cells by inducing apoptosis, ferroptosis, necroptosis, and autophagy [131]. This warrants advanced experiments to explore potential ferroptosis-modulating targets in the future.

### 2.2. Ferroptosis-Inhibiting Natural Products

Many natural products are potential ferroptosis-inhibiting genes (Table 1). Although this review focuses on ferroptosis, some ferroptosis-inhibiting natural products exhibiting ferroptotic and non-ferroptotic effects are mentioned. Some studies on ferroptosis-inhibiting natural products did not examine ferroptosis but showed the impact of ferroptosis due to the modulation of ferroptosis-inhibiting genes.

In general, natural products upregulate ferroptosis-inhibiting genes or downregulate ferroptosis-inducing genes (Figure 2). The ferroptosis-inhibiting natural products and miRNAs were retrieved by a literature search (PubMed and Google Scholar), while the potential targets of ferroptosis-inhibiting RNAs were retrieved from the miRDB database [19]. The ferroptosis-inhibiting functions of reported natural products and their modulation (inducing or inhibiting) on ferroptosis-inducing or ferroptosis-inhibiting targets are exemplified as follows (Table 1). Natural products that modulate specific proteins in the molecular pathway of inhibiting ferroptosis are shown (Figure 3).

#### 2.2.1. Cancer Studies for Ferroptosis-Inhibiting Natural Products

##### Nodosin, Nordihydroguaiaretic Acid, and Cryptotanshinone

The modulating effects of ferroptosis-inhibiting natural products, such as nodosin, nordihydroguaiaretic acid, and cryptotanshinone, are reported in cancer studies (Table 1). Nodosin and nordihydroguaiaretic acid inhibit ferroptosis by regulating ferroptosis-modulating genes. Nodosin inhibits the migration of bladder cancer by inhibiting ferroptosis [142]. Nodosin promotes the expression of the ferroptosis-inhibiting *AIFM2* gene, and in turn, AIFM2 interacts with the ferroptosis-inhibiting GPX4 protein to inhibit lipid peroxidation and ferroptosis [142]. Nordihydroguaiaretic acid, an inhibitor of the ferroptosis-inducing proteins ALOX12/15, alleviates the GPX4 inhibitor (RLS3)-triggered ferroptosis of acute lymphoblastic leukemia cells [143], suggesting nordihydroguaiaretic acid as a ferroptosis inhibitor. Cryptotanshinone, a *Salvia miltiorrhiza*-derived diterpenoid anthraquinone, suppresses erastin-induced ferroptosis and the cell death of pancreatic cancer cells [144]. However, its regulation on ferroptosis-modulating genes is rarely investigated.

#### 2.2.2. Cancer and Non-Cancer Studies for Ferroptosis-Inhibiting Natural Products

The modulating effects of ferroptosis-inhibiting natural products, such as artepillin C, bakuchiol, glycyrrhizin, psoralidin, and baicalein, are reported in both cancer and non-cancer studies (Table 1).

##### Artepillin C and Bakuchiol

Several natural products demonstrate neuroprotective and neuron-related tumor regression effects by downregulating ferroptosis (Table 1). Artepillin C, a natural product derived from Brazilian green propolis, was shown to have anticancer (neurofibromatosis-associated tumors) impacts [195]. For the non-cancer study, artepillin C exhibits neuroprotective effects on mouse hippocampal HT22 cells by downregulating ferroptosis [145]. Bakuchiol, a *Cullen corylifolium*-derived natural product, shows antiproliferative effects on skin cancer cells [196], but the impact of ferroptosis is not assessed. In comparison, the potential ferroptosis of bakuchiol was reported in non-cancer cells. For example, bakuchiol suppresses the erastin-triggered ferroptosis of mouse hippocampal cells [146]. The impact of ferroptosis on these artepillin C and bakuchiol studies needs to be further assessed.

##### Berberine

Berberine studies showing similar ferroptosis modulation (upregulation and downregulation) are exemplified (Table 1). Berberine alleviates erastin and RSL3 (GPX4 inhibitor)-triggered cell death and the ferroptosis of cardiac cells [147]. Berberine exhibits anticancer effects, such as breast cancer cells [197], but the participation of ferroptosis is not assessed.

For non-cancer studies, berberine was reported to exhibit ferroptosis-modulating effects. Berberine suppresses cerebral ischemia–reperfusion-injury-induced ferroptosis by downregulating *ACSL4* and upregulating *SLC7A11* expression [149]. Without assessing ferroptosis, berberine induces non-ferroptosis effects by modulating ferroptosis-inducing and ferroptosis-inhibiting genes (Table 1). Berberine upregulates the ferroptosis-inhibiting gene ceruloplasmin in diabetic rats [150]. Alternatively, berberine may downregulate the ferroptosis-inducing gene. For example, berberine suppresses liver fibrosis by downregulating the ferroptosis-inducing *ATG5* gene [148]. Berberine suppresses apoptosis by upregulating the ferroptosis-inhibiting *NFE2L2* gene [151]. The impact of ferroptosis on these studies needs to be further assessed.

##### Glycyrrhizin, Psoralidin, and Butein

Similar ferroptosis modulation is applied to glycyrrhizin, psoralidin, and butein (Table 1). Glycyrrhizin inhibits ferroptosis in acute liver failure [152]. Glycyrrhizin demonstrates the antiproliferation of liver cancer cells [198], but the impact of ferroptosis was not investigated. Glycyrrhizin upregulates the ferroptosis-inhibiting *GPX4* gene for hypoxic–ischemic brain damage [153]. Glycyrrhizin stimulates the expression of the ferroptosis-inhibiting *SLC40A1* gene in human lung epithelial cells [154].

Psoralidin suppresses the ferroptosis of hippocampal cells based on an erastin-induced ferroptosis-mediated cell viability assay [146]. Psoralidin exerts tumor-growth-suppressing effects based on a breast cancer cell xenografted mouse model [199]. However, the impact of ferroptosis was not assessed.

Butein alleviates the erastin-induced ferroptosis of bone-marrow-derived mesenchymal stem (BMSCs) cells via its antioxidant properties [155]. Notably, butein may regulate non-ferroptosis responses. Butein triggers apoptosis and suppresses the migration of liver cancer cells by upregulating the ferroptosis-inhibiting *SP1* gene [156]. The role of ferroptosis in these non-ferroptosis studies needs to be further validated.

##### Baicalein

Similar ferroptosis modulation is applied to baicalein (Table 1). Baicalein is a ferroptosis inhibitor [200]. For example, baicalein suppresses ferroptosis to decrease cerebral ischemia–reperfusion injury by downregulating the ferroptosis-inducing *ACSL4* gene and upregulating ferroptosis-inhibiting genes (*GPX4* and *SLC7A11*) in brain tissues [157]. Baicalein alleviates carbon-tetrachloride-triggered acute liver injury and ferroptosis in mice by downregulating the ferroptosis-inducing *ALOX12* gene [159]. Moreover, baicalein inhibits the ferroptosis-inducing *ALOX15* gene [160]. Baicalein suppresses ferroptosis and restores the phagocytosis of monocytes by upregulating the ferroptosis-inducing and ferroptosis-inhibiting *GPX4* gene [161].

Without assessing ferroptosis, baicalein induces non-ferroptosis effects by modulating ferroptosis-inducing and ferroptosis-inhibiting genes (Table 1). Baicalein downregulates the palmitate-induced ferroptosis-inducing *ATF3* gene in rat insulinoma cells, avoiding lipotoxicity [158]. Baicalein reduces acetaminophen-induced hepatotoxicity in mice by upregulating the hepatic ferroptosis-inhibiting *GCLC* gene [162]. A detailed evaluation of their ferroptosis response is warranted.

#### 2.2.3. Non-Cancer Studies for Ferroptosis-Inhibiting Natural Products

The modulating effects of ferroptosis-inhibiting natural products are reported in non-cancer studies (Table 1).

Several natural products, such as 7-O-cinnamoyl-taxifolin, 3-hydroxybakuchiol, and Morachalcone D, demonstrate the inhibition of ferroptosis of mouse hippocampal neuronal cells (Table 1). The natural product 7-O-cinnamoyl-taxifolin suppresses the RSL3 (GPX4 inhibitor)-induced ferroptosis of hippocampal neuronal cells by upregulating the ferroptosis-inhibiting *GPX4* gene [163]. The natural product 3-hydroxybakuchiol, derived from *Cullen corylifolium*, is a ferroptosis inhibitor for hippocampal cells based on an erastin-induced ferroptosis cell viability assay [146]. Morachalcone D, a mulberry-leaf-derived prenylated chalcone, inhibits the erastin-induced ferroptosis-based cell death of hippocampal cells by upregulating ferroptosis-inhibiting genes (*SLC7A11*, *NFE2L2*, and *GPX4*) [164]. Moreover, sterubin exerts neuroprotective and anti-inflammatory effects by upregulating the ferroptosis-inhibiting *NFE2L2* gene [165], but its ferroptosis effects need further validation.

Ferroptosis-inhibiting natural products such as puerarin and proanthocyanidin show non-neuroprotection effects (Table 1). Puerarin, a *Pueraria lobata*-derived natural product, attenuates ferroptosis-dependent cardiomyocyte injury [170]. Puerarin inhibits subarachnoid-hemorrhage-induced ferroptosis in rats by upregulating *NFE2L2* and *GPX4* [172]. Moreover, puerarin activates the ferroptosis-inhibiting *GCLC* gene [171]. Proanthocyanidin enhances the locomotion of spinal cord injury mice by downregulating ferroptosis [166]. Proanthocyanidin inhibits ferroptosis and influenza-virus-induced acute lung injury in mice by downregulating *ACSL4* and upregulating *GPX4* and *SLC7A11* [168]. Proanthocyanidin modulates the ferroptosis-inducing and ferroptosis-inhibiting genes without assessing ferroptosis in non-cancer studies. Proanthocyanidins inhibit influenza-virus-induced autophagy by downregulating *ATG7* and *ATG5* [167]. Proanthocyanidins inhibit gluconeogenesis in type 2 DM mice, achieving an anti-diabetes effect, by downregulating DPP4 activity [169]. Consequently, a detailed assessment of the puerarin and proanthocyanidin treatments is warranted in cancer studies.

### 2.3. Exosome Regulation by Ferroptosis-Modulating Natural Products

Among these 55 ferroptosis-modulating natural products, only 9 natural products are also reported in exosome modulation studies. For example, artesunate upregulates exosomal small nucleolar RNA host gene 7 (SNHG7) to enhance osteoblast activity and suppress osteogenesis in mice [201]. Curcumin induces anti-lung-cancer effects by upregulating exosomal transcription factor 21 (TCF21) expression and downregulating DNA (cytosine-5)-methyltransferase 1 (DNMT1), i.e., a TCF21 suppressor [202]. In EGCG treatment, breast cancer exosomes exhibit tumor-suppressive effects by suppressing macrophage M2 polarization [203]. Gallic acid exerts anti-breast-cancer effects by suppressing exosomal secretion [204]. Matrine induces the antiproliferation of colon cancer cells by suppressing exosomal circSLC7A6 secretion from cancer-associated fibroblasts (CAFs) [205]. Quercetin exhibits the antiproliferation of colon cancer cells, accompanied by enriching exosome miRNA amounts [206]. Sulforaphane blocks proliferation and autophagy by inducing exosome-dependent paracrine senescence [207]. Milk-derived exosomes of withaferin-A-treated breast cancer cells have been effectively transferred to target cells for anticancer purposes [208]. Exosomes derived from β-elemene-treated breast cancer cells trigger apoptosis and inhibit chemoresistance, accompanied by downregulating multiple drug-resistant proteins, such as p-glycoprotein [209]. Colon-cancer-cell-isolated exosomes exhibit tumor-promoting effects, which are suppressed by berberine [210].

Detailed investigation of the regulation of exosome biogenesis by the remaining ferroptosis-modulating natural products is warranted. The potential impact on exosome biogenesis by ferroptosis-modulating natural products is discussed later.

## 3. Ferroptosis-Modulating miRNAs and Their Ferroptosis-Targeting Genes

Many miRNAs showing ferroptosis-modulating effects have been well-reviewed [35,36,37]; however, these studies provide one or two ferroptosis-modulating targets for ferroptosis-modulating miRNAs from literature reports. Utilizing miRDB database [19] mining (Figure 2), more ferroptosis-modulating (inducing and inhibiting) genes were retrieved in ferroptosis-modulating miRNA studies, although they did not assess ferroptosis changes (Table 2). Ferroptosis-inducing (Section 3.1) and ferroptosis-inhibiting (Section 3.2) miRNAs and their ferroptosis-modulating targets are illustrated later.

The Google Scholar search methodology for ferroptosis-modulating miRNAs and their target genes is described as follows: Based on the search term “miRNA ferroptosis”, only those miRNAs with identified complete names showing 3p or 5p information are included if available (Table 2). Then, this complete name information for miRNAs is suitable for an miRDB-targeted search for the modulation of ferroptosis bioinformatically.

**Table 2:** Ferroptosis-modulating miRNAs and their ferroptosis-targeting genes.

	Ferroptosis- Modulating miRNA	Cancer Cells	Targets	miRDB-Targeting Ferroptosis-Inducing/ Inhibiting Genes (Targets)
Ferroptosis-inducing miRNAs	miR-1261 [211]	Liver Ca	SLC7A11	
	miR-143-3p [212]	Renal Ca		SLC7A11
	miR-34c-3p [213]	Oral Ca		
	miR-382-5p [214]	Ovarian Ca		
	miR-489-3p [215]	Gastric Ca		
	miR-25-3p [216]	Prostate Ca		SLC7A11, AIFM1, SLC11A2
	miR-409-3p [217]	Cervical Ca		SLC7A11
	miR-515-5p [217]	Cervical Ca		
	miR-545-3p [218]	Thyroid Ca		GCLC, SLC11A2
	miR-27a-3p [219]	Bladder Ca		SLC7A11, NEDD4, NFE2L2
	miR-375-3p [220]	Oral Ca		SLC7A11
	miR-205-5p [221]	Airway epithelial *		TXNRD1
	miR-302a-3p [222]	Lung Ca	SLC40A1	SLC40A1, AIFM1
	miR-4735-3p [223]	Renal Ca		HIF1A, NEDD4, GCLC, SLC40A1
	miR-142-3p [224]	Liver Ca	SLC3A2	SLC7A11
	miR-1231 [225]	Thyroid Ca	GPX4	BGN
	miR-1287-5p [226]	Lung Ca		
	miR-15a-5p [227]	Prostate Ca		SLC11A2
	miR-15a-3p [228]	Colon Ca		HIF1A
	miR-539-5p [229]	Colon Ca		SP1, TXNRD1, SLC11A2, SLC7A11, SLC40A1
	miR-541-3p [230]	Liver Ca		
	miR-324-3p [231]	Lung Ca		SLC7A11
	miR-450b-5p [232]	Liver Ca		NFE2L2, CP, SLC7A11, AIFM1
	miR-125b-5p [233]	Oral Ca	NFE2L2	AIFM1, TXNRD1
	miR-144-3p [234]	Leukemia		NFE2L2, SLC7A11, GCLC
	miR-28-5p [235]	Breast Ca		NFE2L2
	miR-507 [236]	Esophageal Ca		NFE2L2
	miR-29b-1-5p [237]	Breast Ca		PROM2
	miR-365a-3p [238]	Liver Ca		
	miR-214-3p [239]	Liver Ca	ATF4	TFAP2C, GPX4
	miR-3200-5p [240]	Liver Ca		
	miR-1228-3p [241]	Breast Ca	AIFM2	
	miR-429 [29]	Gastric Ca	BGN	
	miR-19b-3p [242]	Lung Ca	FTH1	SLC11A2
	miR-129-5p [243]	Bladder Ca	PROM2	NFE2L2
	miR-101-3p [27]	Lung Ca	TBLR1	NFE2L2, SLC7A11, GCLC
Ferroptosis-inhibiting miRNAs	miR-23a-3p [244]	Liver Ca	ACSL4	EPAS1
	miR-424-5p [245]	Ovarian Ca		ACSL4, YAP1
	miR-4291 [246]	Cervical Ca		DPP4, NCOA4, YAP1
	miR-670-3p [247]	GBM		ACSL4
	miR-18a-5p [248]	GBM	ALOXE3	WWTR1
	miR-522-3p [249]	Gastric Ca	ALOX15	WWTR1, ACSL4
	miR-19a-3p [21]	Colon Ca	IREB2	IREB2, ACSL4, NCOA4, ATG5

Ca: cancer cells. * Non-cancer cells. Ferroptosis-inducing genes (ACSL4, ALOXE3, ALOX15, and IREB2) and ferroptosis-inhibiting genes (SLC7A11, SLC40A1, SLC3A2, GPX4, NFE2L2, ATF4, AIFM2, BGN, FTH1, PROM2, and TBLR1) have been mentioned (Section 1.3). The blank column indicates data are not available by miRDB retrieval.

### 3.1. Ferroptosis-Inducing miRNAs and Their Ferroptosis-Targeting Genes

miRNAs can bind to their target and downregulate target expression. The rationale is that ferroptosis-inducing miRNAs are expected to target ferroptosis-inhibiting genes (Figure 2). Different ferroptosis miRNAs may have the same targets. Many ferroptosis-inducing miRNAs and targets from literature reports and miRDB mining were exemplified in the order of target genes (Table 2).

#### 3.1.1. SLCA11 (Ferroptosis-Inhibiting Gene)

In general, miRNAs that show the downregulation of ferroptosis-inhibiting *SLC7A11* genes are potential ferroptosis-inducing miRNAs (Table 2). Several miRNAs, such as miR-1261, miR-143-3p, miR-34c-3p, miR-382-5p, and miR-489-3p, were reported to induce ferroptosis in cancer cells by targeting and downregulating the ferroptosis-inhibiting *SLC7A11* gene. This has been shown in the ferroptosis induction of liver cancer cells by miR-1261 [211], renal cancer cells by miR-143-3p [212], oral cancer cells by miR-34c-3p [213], ovarian cancer cells by miR-382-5p [214], and gastric cancer cells by miR-489-3p [215].

Some miRNAs show *SLC7A11*-modulating effects, but the evidence of ferroptosis induction is indirect (Table 2). TFAP2C mediated the inhibition of ferroptosis on docetaxel-resistant prostate cancer cells by downregulating miR-25-3p and upregulating miR-25-3p to target *SLC7A11* [216], suggesting that miR-25-3p is a ferroptosis-inducing miRNA. circEPSTI1, which is highly expressed in cervical cancer, inhibits ferroptosis, which downregulates miR-409-3p and miR-515-5p and upregulates *SLC7A11* [217], suggesting miR-409-3p and miR-515-5p are ferroptosis-inducing miRNAs. Similarly, circ_0067934 suppresses ferroptosis and improves the cell viability of thyroid cancer cells by sponging miR-545-3p and upregulating *SLC7A11* [218], indicating that miR-545-3p functions as ferroptosis-inducing miRNA of thyroid cancer cells.

Some miRNAs show *SLC7A11*-modulating effects but lack evidence with respect to ferroptosis induction (Table 2). miR-27a-3p downregulates *SLC7A11* in cisplatin-resistant bladder cancer cells [219]. miR-375-3p inhibits the proliferation of oral cancer cells by downregulating *SLC7A11*, which is reversed by *SLC7A11* overexpression [220]. Moreover, miR-205-5p targets *SLC7A11* based on the dataset for COPD small-airway epithelial cells [221]. This warrants a thoughtful characterization of more ferroptosis responses, such as lipid peroxidation and iron uptake.

For ferroptosis-inducing miRNAs, some reported targets have been retrieved by the miRDB database [19] (Table 2). For example, *SLCA11* is searchable for miR-143-3p, miR-25-3p, miR-27-3p, miR-375-3p, and miR-409-3p by performing an miRDB search. Moreover, some potential ferroptosis-inhibiting genes have not been reported but are retrieved by miRDB.

In addition to *SLCA11*, other potential targets for miR-25-3p are *AIFM1* and *SCL11A2*; *GCLC* and *SLC11A2* are potential targets for miR-27-3p; *NEDD4* and *NFE2L2* are potential targets for miR-545-3p; and *TXNRD1* is a potential target for miR-205-5p (Table 2). All these potential miRDB-targets are ferroptosis-inhibiting genes that are possibly downregulated by these ferroptosis-inducing miRNAs. This warrants a detailed examination of the participation of these ferroptosis-inhibiting genes in studies for ferroptosis-inducing miRNAs.

#### 3.1.2. SLC40A1 and SLC3A2 (Ferroptosis-Inhibiting Genes)

In general, miRNAs showing the downregulation of ferroptosis-inhibiting genes, such as *SLC40A1* and *SLC3A2,* are potential ferroptosis-inducing miRNAs (Table 2). miR-302a-3p and miR-4735-3p trigger the ferroptosis of the lung [222] and renal [223] cancer cells by downregulating *SLC40A1*, respectively. Liver-cancer-cell-derived exosomal miR-142-3p induces ferroptosis in M1-type macrophages by downregulating *SLC3A2* [224].

In addition to *SLC40A1*, another potential miRDB target for miR-302a-3p is *AIFM1*, while those for miR-4735-3p include *HIF1A*, *NEDD4*, and *GCLC* (Table 2). *SLC7A11* is retrieved by performing an miRDB search. These potential miRDB targets are ferroptosis-inhibiting genes that are possibly downregulated by ferroptosis-inducing miRNAs as described. This warrants a detailed assessment of the participation of these ferroptosis-inhibiting genes in these ferroptosis-inducing miRNA experiments.

#### 3.1.3. GPX4 (Ferroptosis-Inhibiting Gene)

In general, miRNAs demonstrating the downregulation of the ferroptosis-inhibiting *GPX4* gene exhibit the potential for being ferroptosis-inducing miRNAs (Table 2). This has been demonstrated in the ferroptosis induction and antiproliferation of papillary thyroid cancer cells by miR-1231 [225], osteosarcoma by miR-1287-5p [226], prostate cancer cells by miR-15a-5p [227], colon cancer cells by miR-15a-3p [228], and colon cancer cells by miR-539-5p [229]. Furthermore, inhibiting miR-541-3p promotes the proliferation and inhibits the ferroptosis of liver cancer cells by upregulating *GPX4* [230], suggesting miR-541-3p is a ferroptosis-inducing miRNA.

Moreover, ferroptosis may attenuate drug resistance, involving miRNAs with *GPX4*-downregulating abilities (Table 2). By downregulating *GPX4* for ferroptosis induction, miR-324-3p attenuates cisplatin resistance in lung cancer cells [231], and miR-450b-5p suppresses sorafenib resistance in liver cancer cells [232].

In addition to *GPX4*, other non-*GPX4* miRDB targets (ferroptosis-inhibiting genes) are retrieved for miR-1231 (*BGN*), miR-15a-5p (*SLC11A2*), miR-15a-3p (*HIF1A*), miR-539-5p (*SP1*, *TXNRD1*, *SLC11A2*, *SLC7A11*, and *SLC40A1*), miR-324-3p (*SLC7A11*), and miR-450b-5p (*NFE2L2*, *CP*, *SLC7A11*, and *AIFM1*) (Table 2). A detailed assessment is warranted to validate the contribution of these miRDB targets to ferroptosis induction.

#### 3.1.4. NFE2L2 (Ferroptosis-Inhibiting Gene)

In general, miRNAs demonstrating the downregulation of the ferroptosis-inhibiting gene *NFE2L2* are potential ferroptosis-inducing miRNAs (Table 2). This has been demonstrated in the cisplatin sensitization of oral cancer cells by miR-125b-5p [233], acute myeloid leukemia by miR-144-3p [234], breast cancer cells by miR-28-5p [235], and esophageal cancer cells by miR-507 [236].

Moreover, natural products and toxins may downregulate *NFE2L2* (Table 2). Parthenolide upregulates miR-29b-1-5p by downregulating *NFE2L2* in breast cancer cells [237]. Cadmium stress induces liver cancer cell death by upregulating miR-365a-3p and downregulating its target *NFE2L2* [238]. Although these miRNAs show *NFE2L2*-modulating ability, this warrants a thoughtful characterization for more ferroptosis responses in these studies in the future.

The reported *NFE2L2* is also searchable for miR-144-3p, miR-28-5p, and miR-507 by miRDB (Table 2). In addition to *NFE2L2*, other potential miRDB targets are retrieved for miR-125b-5p (*AIFM1* and *TXNRD1*), miR-144-3p (*SLC7A11* and *GCLC*), and miR-29b-1-5p (*PROM2*). An advanced assessment to validate their ferroptosis-modulating functions is warranted.

#### 3.1.5. ATF4 (Ferroptosis-Inhibiting Gene)

miRNAs demonstrating the downregulation of the ferroptosis-inhibiting *ATF4* gene are potential ferroptosis-inducing miRNAs (Table 2). With the downregulation of *ATF4*, miR-214-3p promotes erastin-triggered ferroptosis and the cell death of liver cancer cells [239], and miR-3200-5p improves ferroptosis and inhibits the proliferation and metastasis of liver cancer cells [240].

In addition to *NFE2L2*, other potential miRDB targets are retrieved for miR-214-3p (*TFAP2C* and *GPX4*) (Table 2).

#### 3.1.6. AIFM2, BGN, FTH1, PROM2, and TBLR1 (Ferroptosis-Inhibiting Genes)

miRNAs showing the downregulation of ferroptosis-inhibiting genes, such as *AIFM2*, *BGN*, *FTH1*, *PROM2*, and *TBLR1*, are potential ferroptosis-inducing genes (Table 2). miR-1228-3p promotes ferroptosis by targeting the ferroptosis-inhibiting *AIFM2* gene in breast cancer cells [241]. miR-429 causes the ferroptosis of gastric cancer cells by downregulating *BGN* [29]. Curcumenol exerts ferroptosis-promoting effects on lung cancer cells by upregulating miR-19b-3p and downregulating *FTH1* [242]. Downregulating miR-129-5p improves proliferation and suppresses the ferroptosis of bladder cancer cells by upregulating *PROM2* [243]. miR-101-3p is underexpressed in lung cancer tissues and cells used for promoting proliferation, which is reversed by miR-101-3p overexpression restoring the ferroptosis of lung cancer cells by targeting *TBLR1* [27] to downregulate GPX4 [179]. These results suggest that miR-19b-3p, miR-129-5p, and miR-101-3p are ferroptosis-inducing genes.

In addition to *FTH1*, *PROM2*, and *TBLR1*, other potential miRDB targets are retrieved for miR-19b-3p (*SLC11A2*), miR-129-3p (*NFE2L2*), and miR-101-3p (*NFE2L2*, *SLC7A11*, and *GCLC*) (Table 2).

### 3.2. Ferroptosis-Inhibiting miRNAs and Their Ferroptosis-Targeting Genes

miRNAs can bind to their target and downregulate target expression. The rationale is that ferroptosis-inhibiting miRNAs are expected to target ferroptosis-inducing genes (Figure 2). Several ferroptosis-inhibiting miRNAs and targets from literature reports and miRDB mining (Table 2) are exemplified in the order of target genes.

#### 3.2.1. ACSL4 (Ferroptosis-Inducing Gene)

miRNAs showing the downregulation of the ferroptosis-inhibiting *ACSL4* gene have the potential to induce ferroptosis (Table 2). miR-23a-3p, highly expressed in liver cancer cells, inhibits ferroptosis by targeting *ACSL4* [244]. miR-424-5p inhibits ferroptosis and the cell death of ovarian cancer cells by targeting *ACSL4*, which is reversed by *ACSL4* overexpression or miR-424-5p underexpression [245]. CircLMO1 enhances ferroptosis and the antiproliferation of cervical cancer cells by upregulating *ACSL4*, which is reversed by miR-4291 overexpression or *ACSL4* underexpression [246]. This suggests that miR-4291 is a ferroptosis-inhibiting miRNA. Furthermore, miR-670-3p inhibits the ferroptosis of glioblastoma cells by downregulating *ACSL4* [247].

The reported *ACSL4* is also searchable for miR-424-5p and miR-670-3p by miRDB (Table 2). In addition to *ACSL4*, other potential miRDB targets are retrieved for miR-23a-3p (*EPAS1*), miR-424-5p (*YAP1*), and miR-4291 (*DPP4*, *NCOA4*, and *YAP1*).

#### 3.2.2. ALOXE3, IREB2, and ALOX15 (Ferroptosis-Inducing Gene)

miRNAs showing the downregulation of ferroptosis-inhibiting genes, such as *ALOXE3*, *IREB2*, and *ALOX15*, have the potential to induce ferroptosis (Table 2). miR-18a-5p suppresses ferroptosis to improve glioblastoma development by downregulating *ALOXE3* [248]. miR-19a-3p inhibits ferroptosis and promotes the proliferation and migration of colorectal cancer cells by targeting *IREB2* [21]. Cisplatin and paclitaxel enhance miR-522-3p secretion from cancer-associated fibroblasts (CAFs), leading to the suppression of ferroptosis and enhancing chemoresistance on gastric cancer cells by downregulating *ALOX15* [249].

The reported *IREB2* is also searchable for miR-19a-3p using miRDB (Table 2). In addition to *ALOXE3*, *ALOS15*, and *IREB2*, other potential miRDB targets are retrieved for miR-18a-5p (WWTR1), miR-522-3p (*WWTR1* and *ACSL4*), and miR-19a-3p (*ACSL4*, *NCOA4*, and *ATG5*).

## 4. Ferroptosis-Modulating miRNAs and Their Exosome-Biogenesis-Targeting Genes

The ferroptosis-inducing exosomal miRNAs are summarized in Table 2. However, the potential regulations of the exosome biogenesis function of these miRNAs are not investigated in the literature reports listed in Table 2. Using the literature search (PubMed and Google Scholar), the participation of these ferroptosis-inducing (Section 4.1 and Section 4.2) and ferroptosis-inhibiting (Section 4.3 and Section 4.4) exosomal miRNAs in exosome studies were retrieved (Table 3). Similarly, the potential functions of these ferroptosis-inducing and ferroptosis-inhibiting exosomal miRNAs in modulating exosome biogenesis are investigated by performing miRDB, which is a robust database for providing miRNA targets (Table 3). Moreover, several anti-cancer and non-cancer studies have reported the impacts of several ferroptosis-inducing (Section 4.1 and Section 4.2) and ferroptosis-inhibiting (Section 4.3 and Section 4.4) exosomal miRNAs on exosome biogenesis, although they did not assess the ferroptosis-modulating effects. The detailed information on these concerns is mentioned as follows.

The Google Scholar search methodology for ferroptosis-modulating miRNAs and their exosome biogenesis target genes is described as follows: Ferroptosis-modulating miRNAs listed in Table 2 were combined with “exosome” for the search to obtain the results for “exosomal miRNA studies” (Table 3). Then, this complete name information for miRNAs was suitable for an miRDB-targeted search for the modulation of exosome biogenesis bioinformatically.

**Table 3:** Ferroptosis-modulating miRNAs and their exosome-biogenesis-targeting genes.

	Ferroptosis- Modulating miRNA	Exosomal miRNA Studies	Exosome Biogenesis Genes (miRDB)
Ferroptosis-inducing miRNAs	miR-101-3p [27]	Medulloblastoma [250]	RAB27A
	miR-1231 [225]	Pancreatic ca [251]	
	miR-1287-5p [226]	Inflammatory injury [252]	RAB7A
	miR-129-5p [243]	Colon ca [253]	VPS4B, ATP9A, PDCD6IP
	miR-142-3p [224]	Retinoblastoma [254]	STAM, HGS
	miR-143-3p [212]	Lung ca [255], pancreatic ca [256]	RAB7A
	miR-144-3p [234]	Endothelial cells [257]	VPS4B, PDCD6IP, SMPD3
	miR-15a-5p [227]	Endometrial ca [258], lung ca [259]	MYO5B, VPS4A
	miR-15a-3p [228]	Wound repair [260]	
	miR-19b-3p [242]	Lung ca [261]	SDC1, VPS4B, MYO5B
	miR-28-5p [235]	Lung injury [262]	SDC1
	miR-29b-1-5p [237]		COPS5
	miR-302a-3p [222]	Preeclampsia [263]	SDC1, RAB11A
	miR-3200-5p [240]		SMPD3
	miR-324-3p [231]		RAB7B
	miR-34c-3p [213]	Lung ca [264]	CD34
	miR-365a-3p [238]		MYO5B
	miR-409-3p [217]	Mast cells [265]	STAM
	miR-450b-5p [232]	Rat [266]	ATP9A, RAB11A, PDCD6IP
	miR-507 [236]		RAB11A, STEAP3, PDCD6IP
	miR-515-5p [217]		RAB11A
	miR-539-5p [229]	Stem cells [267]	STAM
	miR-545-3p [218]		RAB11A
Ferroptosis- inhibiting miRNAs	miR-18a-5p [248]	Osteoblast cells [268]	
	miR-19a-3p [21]	Ischemic myocardium [269]	SDC1, VPS4B, MYO5B
	miR-23a-3p [244]	Cholangiocarcinoma [270]	
	miR-424-5p [245]	Endothelial cells [271]	VPS4A, MYO5B
	miR-4291 [246]		ATP9A, SMPD3, TSG101, MYO5B
	miR-522-3p [249]		PDCD6IP
	miR-670-3p [247]		CD34, RAB27A

Exosome-biogenesis-modulating (inducing and inhibiting) genes have been mentioned (Section 1.2). ca = cancer cells. The blank column indicates data are not available by Google Scholar and miRDB retrieval.

### 4.1. The Potential Role of the Exosome Biogenesis Modulation of Ferroptosis-Inducing miRNA in Cancer Studies

#### 4.1.1. Anticancer Effects of Ferroptosis-Inducing miRNAs

Several ferroptosis-inducing miRNAs (Table 2) were reported to regulate exosome biogenesis and contribute to anti-cancer effects (Table 3). Based on cell and animal models, exosomal miR-101-3p suppresses medulloblastoma growth [250]. Exosomal miRNA-1231 derived from bone marrow mesenchymal stem cells inhibits the proliferation and migration of pancreatic cancer cells [251]. Exosomal miR-129-5p inhibits the proliferation and migration of colon cancer cells [253]. Monocyte-derived exosomal miR-142-3p induces the antiproliferation of retinoblastoma cells [254]. Exosomal miR-143-3p is more highly expressed in human mesenchymal stem cells (hMSCs) than in pancreatic cancer cells. Exosomal miR-143-3p inhibits proliferation and triggers the apoptosis of pancreatic cancer cells [256]. Apelin (APLN), a tumor promoter in tumors, inhibits lung cancer cell proliferation by suppressing exosomal miR-15a-5p expression [259], suggesting that exosomal miR-15a-5p has a tumor suppressor function. Moreover, low exosomal miR-34c-3p enhances the migration of lung cancer cells [264], indicating that exosomal miR-34c-3p exhibits a tumor suppressor function.

#### 4.1.2. miRDB Targets of Ferroptosis-Inducing miRNAs

For cancer studies, the miDRB-targeted exosome biogenesis genes for ferroptosis-inducing miRNAs are retrieved as follows (Table 3): miR-101-3p (*RAB27A*), miR-129-5p (*VPS4B*, *ATP9A*, and *PDCD6IP*), miR-142-3p (*STAM* and *HGS*), miR-143-3p (*RAB7A*), miR-144-3p (*VPS4B*, *PDCD6IP*, and *SMPD3*), miR-15a-5p (*MYO5B* and *VPS4A*), miR-19b-3p (*SDC1*, *VPS4B*, and *MYO5B*), miR-29b-1-5p (*COPS5*), miR-3200-5p (*SMPD3*), miR-324-3p (*RAB7B*), miR-34c-3p (*CD34*), miR-365a-3p (*MYO5B*), miR-507 (*RAB11A*, *STEAP3*, and *PDCD6IP*), miR-515-5p (*RAB11A*), and miR-545-3p (*RAB11A*). These predicted exosome biogenesis targets support the potential exosome-modulating effects for cancer cells, as reported (Table 3).

Some miDRB-targeted exosome biogenesis genes are shared with several ferroptosis-inducing miRNAs. *RAB11A* is targeted by miR-507, miR-515-5p, and miR-545-5p. *VPS4B* is targeted by miR-129-5p, miR-144-3p, and miR-19b-3p. *MYO5B* is targeted by miR-19b-3p and miR-365a-3p. *PDCD6IP* is targeted by miR-129-5p, miR-144-3p, and miR-507.

### 4.2. The Potential Role of the Exosome Biogenesis Modulation of Ferroptosis-Inducing miRNA in Non-Cancer Studies

#### 4.2.1. Non-Cancer Functions of Ferroptosis-Inducing miRNAs

Several ferroptosis-inducing miRNAs (Table 2) were reported to regulate exosome biogenesis and modulate non-cancer functions, such as inflammatory, diabetes, organ injury, and angiogenesis responses, as follows (Table 3).

In inflammatory responses, several exosomal miRNAs, such as miR-1287-5p, miR-409-3p, and miR-539-5p, have been reported. Exosomal miR-1287-5p isolated from microscopic polyangiitis plasma can be taken up by human umbilical vein endothelial cells (HUVEC). Subsequently, exosomal miR-1287-5p upregulates inflammatory and adhesion factors and promotes neutrophil adhesion, which is reversed by downregulating miR-1287-5p [252]. Exosomal miR-409-3p released from lipopolysaccharide-stimulated mast cells activates microglial mobility and neuroinflammation [265]. Furthermore, the exosomal miR-539-5p of bone marrow mesenchymal stem cells suppresses pyroptosis. It decreases proinflammatory cytokines, thereby attenuating inflammatory bowel disease by targeting and downregulating the pyroptotic protein, NLR family pyrin domain containing 3 (NLRP3) [267].

In diabetes response, several exosomal miRNAs, such as miR-144-3p and miR-15a-3p, have been reported. Circulating exosomal miR-144-3p, derived from mice with streptozotocin-induced diabetes, inhibits the migration of endothelial progenitor cells and ischemia-triggered neovascularization [257]. Plasma exosomal miR-15a-3p from diabetic patients shows inhibitory effects on diabetic wound repair, which are reversed by miR-15a-3p knockdown [260].

In organ injury, several exosomal miRNAs, such as miR-28-5p and miR-450b-5p, have been reported. Mesenchymal stem cells can protect against acute lung injury (ALI). ALI exosomal miR-28-5p from a phosgene-stimulated lung enhances the proliferation and immunomodulation of mesenchymal stem cells [262]. Exosomal miR-450b-5p in plasma shows similar levels of miR-450b-5p as cerebrospinal fluid. Plasma exosomal miR-450b-5p was validated to be a promising biomarker for transient ischemia in rats [266].

Some exosomal miRNAs are reported to modulate angiogenesis. For example, the migration and proliferation of trophoblasts are improved, and the angiogenesis of HUVEC is inhibited, by exosomal miR-302a [263].

#### 4.2.2. miRDB Targets of Ferroptosis-Inducing miRNAs

For non-cancer studies, the miDRB-targeted exosome biogenesis genes for ferroptosis-inducing miRNAs are retrieved as follows (Table 3): miR-1287-5p (*RAB7A*), miR-28-5p (*SDC1*), miR-302a-3p (*SDC1* and *RAB11A*), miR-409-3p (*STAM*), miR-450b-5p (*ATP9A*, *RAB11A*, and *PDCD6IP*), miR-450b-5p (*ATP9A*, *RAB11A*, and *PDCD6IP*), and miR-539-5p (*STAM*). These predicted exosome biogenesis targets of ferroptosis-inducing miRNAs support the potential exosome-modulating effects for non-cancer cells, as reported (Table 3).

Some ferroptosis-inducing miRNAs, such as miR-29b-1-5p, miR-3200-5p, miR-324-3p, miR-365a-3p, miR-507, miR-515-5p, and miR-545-3p, showing the modulation of exosome biogenesis, are rarely investigated based on the PubMed and Google Scholar search. This review fills the gap by performing the miRDB search. The miRDB-targeted exosome biogenesis genes for these ferroptosis-inducing miRNAs are retrieved as follows (Table 3): miR-29b-1-5p (*COPS5*), miR-3200-5p (*SMPD3*), miR-324-3p (*RAB7B*), miR-365a-3p (*MYO5B*), miR-507 (*RAB11A*, *STEAP3*, and *PDCD6IP*), miR-515-5p (*RAB11A*), and miR-545-3p (*RAB11A*).

### 4.3. The Potential Role of the Exosome Biogenesis Modulation of Ferroptosis-Inhibiting miRNA in Cancer Studies

Several ferroptosis-inhibiting miRNAs, such as miR-18a-5p and miR-23a-3p, can exhibit exosome-associated regulation, as shown in cancer studies (Table 3). miR-18a-5p is overexpressed in the bone metastases of prostate cancer patients. The inhibition of prostate-cancer-cell-derived exosomal miR-18a-5p suppresses sclerotic lesions from bone metastases in mice [268]. miR-23a-3p is highly expressed in cholangiocarcinoma and promotes proliferation, which is reversed by miR-23a-3p knockdown. The exosomal miR-23a-3p of cholangiocarcinoma improves proliferation and metastasis [270].

### 4.4. The Potential Role of the Exosome Biogenesis Modulation of Ferroptosis-Inhibiting miRNA in Non-Cancer Studies

Several ferroptosis-inhibiting miRNAs, such as miR-19a-3p and miR-424-5p, can regulate exosome-associated non-cancer diseases. For example, shock wave therapy can improve the functions of myocardial ischemia, such as angiogenesis and anti-myocardial fibrosis, by releasing angiogenic exosomal miR-19a-3p [269]. Exosomal miR-424-5p from oxygen–glucose-deprivation-activated microglia enhances the injury of brain microvascular endothelial cells and inhibits their vascular formation, which is reversed by miR-424-5p knockdown [271].

For non-cancer studies, the miDRB-targeted exosome biogenesis genes for ferroptosis-inhibiting miRNAs are retrieved as follows (Table 3): miR-19a-3p (*SDC1*, *VPS4B*, and *MYO5B*), miR-424-5p (*VPS4A* and *MYO5B*), miR-4291 (*ATP9A*, *SMPD3*, *TSG101*, and *MYO5B*), miR-522-3p (*PDCD6IP*), and miR-670-3p (*CD34* and *RAB27A*). As reported, these predicted exosome biogenesis targets of ferroptosis-inhibiting miRNAs support the potential exosome-modulating effects of non-cancer cells (Table 3).

Some ferroptosis-inhibiting miRNAs, such as miR-4291, miR-522-3p, and miR-670-3p, modulating exosome biogenesis are rarely investigated based on PubMed and Google Scholar searches. This gap is filled by performing an miRDB search. The miRDB-targeted exosome biogenesis genes for these ferroptosis-inhibiting miRNAs are retrieved as follows (Table 3): miR-4291 (*ATP9A*, *SMPD3*, *TSG101*, and *MYO5B*), miR-522-3p (*PDCD6IP*), and miR-670-3p (*CD34* and *RAB27A*).

## 5. Ferroptosis-Modulating miRNAs Are Associated with Some Natural Products

Several natural products (Table 1) and miRNAs (Table 2) exhibiting ferroptosis-modulating effects have been individually reported as described above. However, the relationship between these ferroptosis-modulating natural products and miRNAs is rarely investigated. This gap is filled by the literature search using Google Scholar. The results are exemplified in the order of these ferroptosis-inducing (Section 5.1, Section 5.2, Section 5.3, Section 5.4 and Section 5.5) and ferroptosis-inhibiting (Section 5.6) miRNAs as follows (Table 4). Finally, the natural-product-centric overview connecting to miRNAs is explored (Section 5.7).

Notably, the literature results (Table 4) demonstrate that these natural products (Table 1) are modulated by various miRNAs (Table 2). However, their impacts on the modulation of ferroptosis have not yet been reported. A detailed investigation of ferroptosis responses for these natural products and miRNAs is warranted.

**Table 4:** The connection between ferroptosis-modulating miRNAs and natural products.

	miRNAs	Ferroptosis-Modulating Natural Products
Ferroptosis-inducing miRNAs	miR-101-3p	Curcumin [272]
	miR-125b-5p	PEITC [273], quercetin [274], EGCG [275], berberine [276]
	miR-1287-5p	Curcumin [277]
	miR-129-5p	Matrine [278]
	miR-142-3p	Artesunate [279], quercetin [280], curcumin [281]
	miR-143-3p	Curcumin [282], EGCG [283,284], sulforaphane [285], quercetin [286]
	miR-144-3p	Curcumin [287]
	miR-15a-5p	Curcumin [288], baicalin [289], Withaferin A [290]
	miR-15a-3p	EGCG [284]
	miR-205-5p	Cryptotanshinone ↓ [291] proanthocyanidins ↓ [292], curcumin [293]
	miR-214-3p	EGCG [284], sulforaphane [285]
	miR-25-3p	Withaferin A [294]
	miR-27a-3p	β-elemene [295], quercetin [296]
	miR-28-5p	Curcumin [297]
	miR-302a-3p	Curcumin [298]
	miR-324-3p	Salinomycin ↓ [299]
	miR-365a-3p	Sulforaphane [300]
	miR-375-3p	Solasonine [301]
	miR-409-3p	Curcumin [302]
	miR-429	Curcumin [303], berberine ↓ [304,305]
	miR-489-3p	Curcumin [306]
Ferroptosis- inhibiting miRNAs	miR-18a-5p	Curcumin ↓ [307]
	miR-19a-3p	Berberine [308], proanthocyanidins [292], matrine ↓ [309], Sulforaphane ↓ [310]
	miR-23a-3p	Berberine [311]
	miR-424-5p	Curcumin [312]
	miR-522-3p	EGCG ↓ [284]

Listed natural products upregulate most miRNAs except for the symbol ↓, which indicates miRNA downregulation. Ferroptosis-modulating natural products and miRNAs have been mentioned in Table 1 and Table 2 and were independently reported in different studies. The references shown in Table 3 provide findings that these natural products can modulate these miRNAs. However, these studies did not report on the changes in ferroptosis.

### 5.1. Ferroptosis-Inducing miRNAs: miR-101-3p, miR-125b-5p, miR-1287-5p, and miR-129-5p

Several ferroptosis-inducing natural products (Table 1) are connected to ferroptosis-inducing miRNAs (Table 3) by carrying out a literature search using PubMed and Google Scholar. The connecting results are summarized (Table 4).

Several ferroptosis-inducing natural products, such as curcumin and matrine, are correlated with the upregulation of the ferroptosis-inducing miRNAs, such as miR-101-3p, miR-1287-5p, and miR-129-5p (Table 4). Curcumin, a ferroptosis inducer, inhibits the cell proliferation of colon cancer cells by upregulating the ferroptosis-inducing miR-101-3p [272]. Similarly, ischemic stroke patients contain low levels of miR-1287-5p. Curcumin may alleviate ischemic stroke by suppressing the oxygen–glucose deprivation/reperfusion (OGD/R)-downregulated miR-1287-5p in neuroblastoma cells [277]. Consequently, curcumin upregulates miR-1287-5p to protect against ischemic stroke. Matrine suppresses lipopolysaccharide (LPS)-triggered epithelial–mesenchymal transition (EMT) in human peritoneal mesothelial cells (HPMCs) by upregulating miR-129-5p [278].

Several ferroptosis-inducing natural products, such as PEITC, quercetin, and EGCG, are correlated with the upregulation of the ferroptosis-inducing miR-125b-5p (Table 4). PEITC induces miR-125b-5p in environmental-cigarette-smoke-exposed mice [273]. Quercetin-enriched high-fat diets promote miR-125b-5p expression in mice [274]. EGCG improves cognitive function in mice models with early-onset Alzheimer’s disease by upregulating circulating exosomal miR-125b-5p [275]. In contrast, ferroptosis-inhibiting natural products, such as berberine, are correlated with the downregulation of the ferroptosis-inducing miR-125b-5p. The ferroptosis inhibitor berberine inhibits the expression of the miR-99a-125b cluster (miR-99a, let-7c, and miR-125b-5p), inducing the apoptosis of multiple myeloma [276].

### 5.2. Ferroptosis-Inducing miRNAs: miR-142-3p, miR-143-3p, miR-144-3p, and miR-15a-5p

Several ferroptosis-inducing natural products, such as artesunate, quercetin, and curcumin, are correlated with the upregulation of ferroptosis-inducing miR-142-3p (Table 4). Artesunate induces the apoptosis of ovarian cancer by promoting Th1 differentiation and upregulating miR-142-3p [279]. Quercetin inhibits pancreatic cancer cell proliferation by upregulating miR-142-3p [280]. Curcumin suppresses the proteasome and proliferation activity of breast cancer cells by upregulating miR-142-3p to target *PSMB5*, reversed by downregulating miR-142-3p [281].

Similarly, ferroptosis-inducing natural products, such as curcumin and quercetin, are associated with upregulating ferroptosis-inducing miR-143-3p (Table 4). Curcumin inhibits autophagy and enhances the radiosensitivity of prostate cancer cells by upregulating miR-143-3p [282]. EGCG inhibits the proliferation of preadipocytes [283] and breast cancer cells [284] by upregulating miR-143-3p. Additionally, gastric cancer cells express low levels of miR-143-3p. miR-143-3p inhibits autophagy to improve the quercetin-induced antiproliferation of gastric cancer cells [286]. Consequently, miR-143-3p provides a positive regulation of the antiproliferative effects of quercetin.

Other ferroptosis-inducing natural products, such as curcumin and withaferin A, are associated with upregulating ferroptosis-inducing miRNAs (Table 4). Curcumin suppresses the H_2_O_2_-induced apoptosis of cardiomyocytes by upregulating miR-144-3p [287]. Wilms’ tumor 1 (WT1) is highly expressed in leukemia. Curcumin downregulates Wilms’ tumor 1 (WT1) of leukemic cells by upregulating miR-15a-5p, which is reversed by miR-15a-5p inhibition [288]. For withaferin A, it upregulates miR-15a-5p in breast cancer cells [290].

### 5.3. Ferroptosis-Inducing miRNAs: miR-15a-3p, miR-205-5p, miR-214-3p, and miR-27a-3p

Several ferroptosis-inducing natural products, such as salinomycin, sulforaphane, EGCG, and β-elemene, are correlated with the upregulation of the ferroptosis-inducing miRNAs (Table 4). Salinomycin induces the antiproliferation, apoptosis, and EMT expression of head and neck cancer cells by upregulating miR-15a-3p [299]. Sulforaphane reduces cancer stemness and cisplatin resistance of lung cancer cells by upregulating miR-214-3p [285]. EGCG inhibits the proliferation of breast cancer cells by upregulating miR-214-3p [284]. β-elemene suppresses the oxygen-stimulated retinal neovascularization of mice by upregulating miR-27a-3p [295].

Ferroptosis-inducing natural products such as curcumin, cryptotanshinone, and proanthocyanidins may be associated with upregulating ferroptosis-inducing miRNAs (Table 4). Curcumin intake upregulates miR-205-5p in murine melanoma [293]. In comparison, some natural products, such as cryptotanshinone and proanthocyanidins, may be associated with downregulating ferroptosis-inhibiting miRNAs. Cryptotanshinone suppresses the invasion of lung cancer cells by downregulating miR-205-5p [291]. Grape seed proanthocyanidins downregulate the miR-205-5p of colon cancer in mice [292].

### 5.4. Ferroptosis-Inducing miRNAs: miR-28-5p, miR-302a-3p, miR-365a-3p, miR-375-3p, miR-429, and miR-489

Ferroptosis-inducing natural products, such as curcumin, sulforaphane, and solasonine, are associated with upregulating ferroptosis-inducing miRNAs (Table 4). Curcumin induces the antiproliferation and apoptosis of large B-cell lymphoma cells by upregulating miR-28-5p, which is reversed by miR-28-5p inhibition [297]. Similarly, curcumin inhibits the proliferation and EMT gene expression of colon cancer cells by inducing miR-302a-3p expression [298]. Sulforaphane induces apoptosis and inhibits pancreatic tumor growth by upregulating miR-365a-3p [300]. Solasonine inhibits the proliferation of liver cancer cells by upregulating miR-375-3p [301]. Curcumin inhibits the cell proliferation of colon cancer cells by upregulating miR-429 [272]. Curcumin alleviates glucose-fluctuation-promoted renal injury and inhibits Warburg effects by upregulating miR-489-3p [306].

### 5.5. Ferroptosis-Inducing miRNAs: miR-382-5p and miR-409-3p

Ferroptosis-inducing natural product curcumin may enhance drug sensitivity by upregulating ferroptosis-inducing miRNAs (Table 4). Compared to parent cells, the oxaliplatin resistance of colorectal cancer cells is enhanced by downregulating miR-409-3p. Accordingly, curcumin reduces oxaliplatin resistance by targeting excision repair cross-complementing 1 (ERCC1) expression and upregulating miR-409-3p [302].

In comparison, the ferroptosis-inhibiting natural product berberine is associated with downregulating ferroptosis-inducing miRNAs (Table 4). miR-429 is highly expressed in colon cancer. Compared to normal tissues, berberine downregulates miR-429 in colon tumors [305]. Berberine suppresses the proliferation and migration of endometrial stromal cells by decreasing miR-429 expression, which is reversed by miR-429 overexpression [304].

### 5.6. Ferroptosis-Inhibiting miRNAs: miR-18a-5p, miR-19a-3p, miR-23a-3p, and miR-552-3p

Ferroptosis-inducing natural products, such as curcumin, EGCG, and matrine, are associated with downregulating ferroptosis-inhibiting miRNAs (Table 4). Curcumin triggers the apoptosis of RT4 schwannoma cells by downregulating miR-18a-5p [307]. EGCG downregulates miR-552-3p in breast cancer cells [284]. Moreover, matrine alleviates preeclampsia by enhancing trophoblast invasion and suppressing inflammation, accompanied by the downregulation of miR-19a-3p [309].

Ferroptosis-inhibiting natural products, such as berberine and proanthocyanidins, are associated with upregulating ferroptosis-inhibiting miRNAs (Table 4). Berberine raises the level of miR-19a-3p and lowers the level of TF, thus activating MAPK signaling and leading to the apoptosis of cancer cells [308]. The ferroptosis inhibitor berberine induces the G2/M arrest and tumor regression of liver cancer cells by upregulating miR-23a-3p, which is reversed by miR-23a-3p inhibition [311]. Proanthocyanidins are generally isolated from grape seeds. Grape seed proanthocyanidins suppress azoxymethane-promoted colon tumorigenesis in mice by upregulating miR-19a-3p [292].

### 5.7. Natural-Product-Centric Overview Connecting to Ferroptosis-Modulating miRNAs

**Figure 4:**
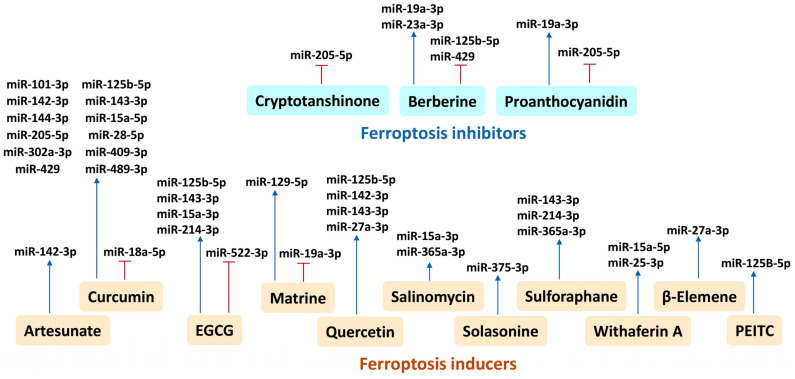
Natural-product-centric overview connecting with ferroptosis-modulating miRNAs. Ferroptosis-modulating natural products and miRNAs are mentioned in Table 1 and Table 2.

Ferroptosis-modulating natural products (Table 1) and miRNAs (Table 2) were independently reported by different studies. Their relationship was summarized in a miRNA-centric manner (Table 4). Alternatively, the natural-product-centric overview to connect with ferroptosis-inducing and ferroptosis-inhibiting miRNAs is summarized (Figure 4).

For ferroptosis-inducing natural products, several natural products, such as curcumin, EGCG, and matrine, may participate in the modulation of multiple miRNAs in different studies. For example, ferroptosis-inducing curcumin upregulates 12 ferroptosis-inducing miRNAs: miR-101-3p [272], miR-125b-5p [277], miR-142-3p [281], miR-143-3p [282], miR-144-3p [287], miR-15a-5p [288], miR-205-5p [293], miR-28-5p [297], miR-302a-3p [298], miR-409-3p [302], miR-429 [303], and miR-489-3p [306]. It downregulates one ferroptosis-inhibiting miRNA (miR-18a-5p [307]). Similarly, EGCG upregulates four ferroptosis-inducing miRNAs (miR-125b-5p [275], miR-143-3p [283,284], miR-15a-3p [284], and miR-214-3p [284]) and downregulates one ferroptosis-inhibiting miRNA (miR-522-3p [284]). Matrine upregulates ferroptosis-inducing miRNA (miR-129-5p) [278] and downregulates ferroptosis-inhibiting miRNA (miR-19a-3p) [309].

Furthermore, one or several ferroptosis-inducing miRNAs are upregulated by the remaining natural products, including artesunate, cryptotanshinone, quercetin, salinomycin, solasonine, sulforaphane, withaferin A, β-elemene, and PEITC (Figure 4).

As for ferroptosis-inhibiting natural products, several natural products, such as cryptotanshinone, berberine, and proanthocyanidin, may contribute to the modulation of multiple miRNAs in different studies. For example, ferroptosis-inhibiting cryptotanshinone upregulates the ferroptosis-inhibiting miRNA (miR-205-5p [291]). Ferroptosis-inhibiting berberine upregulates ferroptosis-inhibiting miRNAs (miR-19a-3p [308] and miR-23a-3p [311]) and downregulates ferroptosis-inducing miRNAs (miR-125b-5p [276] and miR-429 [304,305]). Proanthocyanidin upregulates the ferroptosis-inhibiting miRNA (miR-19a-3p [292]) and downregulates the ferroptosis-inducing miRNA (miR-205-5p) [292].

Consequently, these results indicate that ferroptosis-inducing natural products may upregulate ferroptosis-inducing miRNAs and/or downregulate ferroptosis-inhibiting miRNAs (Figure 2). In contrast, ferroptosis-inhibiting natural products may upregulate ferroptosis-inhibiting miRNAs and/or downregulate ferroptosis-inducing miRNAs.

## 6. Conclusions

Ferroptosis and exosome biogenesis can regulate cell physiological responses in cancer and non-cancer cells. Ferroptosis has the potential to avoid the drug-induced apoptosis resistance of cancer cells [313]. Modulating ferroptosis and exosome biogenesis is a novel strategy for cancer and non-cancer therapies. Natural products are rich resources when applied to cancer and other disease therapies involving the modulation of ferroptosis. Moreover, miRNAs are important in regulating ferroptosis and exosome biogenesis. Therefore, this review proposes a rationale that ferroptosis-modulating natural products may regulate ferroptosis-modulating miRNAs, which in turn control ferroptosis- and exosome-biogenesis-modulating targets.

For the literature search, ferroptosis-modulating natural products (Table 1) and miRNAs (Table 2) were individually reported. However, there is a knowledge gap with respect to the connection to the modulating effects of the miRNA of natural products acting on ferroptosis. There is another knowledge gap with respect to the limited reports on ferroptosis- and exosome-biogenesis-modulating targets of natural-product-regulated miRNAs. Those two gaps are innovatively connected in this review, providing a systemic integration for natural-product-modulated miRNAs and their potential targeting for ferroptosis and exosome biogenesis. To fill these gaps, we used literature-search-derived ferroptosis-modulating miRNAs that were added to the miRDB database for identifying the potential targets of ferroptosis- (Table 2) and exosome-biogenesis-modulating genes (Table 3). Moreover, the connection between natural products and miRNAs regarding their ferroptosis induction was organized (Table 4 and Figure 4).

The central concept of this review clearly and innovatively illustrates that ferroptosis modulation may regulate ferroptosis-modulating miRNAs and target their potential genes for regulating ferroptosis and exosome biogenesis in an integrated scope. In other words, ferroptosis-inducing natural products may upregulate ferroptosis-inducing miRNAs and/or downregulate ferroptosis-inhibiting miRNAs. In turn, they target ferroptosis-inhibiting genes and/or ferroptosis-inducing genes. The ferroptosis-inhibiting natural products show the opposite responses.

Notably, this review provides a straightforward literature survey with insufficient evidence of critical assessments for ferroptosis and exosome biogenesis targeting by natural products. Although miRDB is an authoritative and evidence-based miRNA target prediction database, this information may be based on specific cell lines and treatments. It may be differentially expressed or target different cases. When applied to other therapies, a careful assessment of this miRNA targeting information is warranted. Moreover, the connection between natural products and miRNAs is organized by a literature search without validating ferroptosis responses in these reported studies. A detailed assessment of ferroptosis responses, including iron uptake, membrane lipid peroxidation, and ferroptosis signaling, for these natural products and miRNAs is warranted.

Moreover, the ferroptosis-modulating effects of the natural products mentioned in the review are not the sole reason for regulating cancer- and non-cancer-cell responses. Other non-ferroptosis effects are also reported in some of those literature reports. Additionally, this review only focuses on exploring the impact of ferroptosis on exosome biogenesis regarding ferroptosis-modulating natural products and miRNAs, whereas the molecular mechanisms by which exosomes may induce ferroptosis need to be investigated in the future.

Consequently, the connections in the natural product–miRNA–ferroptosis–exosome biogenesis axis are well organized. This review sheds light on the potential directions for integrating miRNAs, exosome biogenesis, and ferroptosis-modulated effects with therapies for cancer and other diseases via natural products.

## Data Availability

No new data were created.
